# Behavioral, neuronal, and physiological facets of multidimensional body image in anorexia nervosa: a scoping review

**DOI:** 10.1186/s40337-025-01191-4

**Published:** 2025-02-10

**Authors:** Monica Di Giuliano, Feliberto de la Cruz, Andy Schumann, Regine Zopf, Karl-Jürgen Bär

**Affiliations:** 1 Lab for Autonomic Neuroscience, Imaging and Cognition (LANIC), Department of Psychosomatic Medicine and Psychotherapy, Jena University Hospital, Krautgasse 8, 07743 Jena, Germany; 2 Department of Cognitive Science, Macquarie University, Sydney, Australia

**Keywords:** Anorexia nervosa, Body image, Body image perception dimension, Body image emotion dimension, Body image cognitive dimension, Body image somato-sensory dimension

## Abstract

Body image is a core identity facet in anorexia nervosa (AN). Current literature outlines body image as a multidimensional construct, characterized by perceptual, cognitive, and emotional components, which explain how individuals perceive, think, and behave in relation to their body. The purpose of this scoping review is to uncover neuropsychological disturbances in AN related to body image by focusing on its different behavioral and neuronal assessments, aiming to provide a broad overview of common findings and limitations. Additionally, we explore the role of somato-sensory/somato-motor components and physiological markers underlying body image disturbances, which have not been extensively examined in previous studies. Literature screening was performed across three databases (PubMed, PsycINFO, Scopus). We classified behavioral, physiological, and neuroimaging studies according to their degree of involvement with body image dimensions and discussed the primary findings for each component. A total of 4015 studies were identified, of which 107 were eligible (84 behavioral and 23 neuroimaging studies) based on eligibility criteria, covering research from 1973 to 2024. Notably, of those which specified the following factors: 16 studies focused on the restrictive subtype (AN-r), two on the binge-eating/purging subtype (AN-b/p), and 19 examined both AN-r and AN-b/p; finally four on the atypical subtype; 11 on the weight-recovered state (WR) and three included also male participants. The mean age across all studies was 20.1 years (SD = 3.79). Our findings reveal that body image disturbances in AN are not limited to the overestimation of body size but also encompass cognitive, emotional, and somato-sensory aspects. A strong drive for thinness emerged as a prominent cognitive factor in maintaining the disturbance, along with altered autobiographical recall, executive function, and attentional biases toward body-related stimuli. Emotional regulation difficulties, altered interoceptive awareness, and distorted somato-sensory aspects—reflected in tactile and motor processes—were highlighted. In addition, increased autonomic reactivity to body image stimuli, indicating a sympathetic/parasympathetic imbalance, is supported by specific cortical-subcortical functional alterations. Our review emphasizes that body image distortion in AN should not be viewed merely as a perceptual deficit but rather as a complex cognitive-emotional and somato-sensory process.

**Supplementary Information:**

The online version contains supplementary material available at 10.1186/s40337-025-01191-4.

## Abstract (plain language summary)

Body image plays a central role in anorexia nervosa (AN), shaping how individuals perceive, think, and feel about their bodies. In this review, we examined behavioral, physiological, and neuroimaging studies that explored the perceptual, cognitive, emotional, and sensorimotor aspects of body image in AN. Our findings reveal that body image disturbances in AN extend beyond overestimating body size and weight. Key issues include a strong drive for thinness, distorted attention and memory processes related to body image, and difficulties with emotional regulation when processing body-related stimuli. Altered body awareness, such as distorted sensations of touch and movement, also emerged as a critical factor. This review emphasizes that body image in AN is not solely a "pure" perceptual issue. Instead, it involves complex interactions between cognitive, emotional, and sensorimotor processes. A deeper understanding of these facets can help identify factors that contribute to the development and maintenance of AN and improve treatment approaches.

**Supplementary Information:**

The online version contains supplementary material available at 10.1186/s40337-025-01191-4.

## Background

The way body weight or shape is experienced is one of the key diagnostic criteria of anorexia nervosa disorder [2]. Anorexia nervosa (AN) is a severe and pervasive mental disorder, which primarily affects adolescent girls and young women. The other main DSM-5 diagnostic criteria are restriction of energy intake as well as intense fear of gaining weight or becoming fat [2]. This mental disorder is further classified in two subtypes: restrictive type (AN-r) and binge eating/purging type (AN-b/p), with four levels of severity according to BMI [2, 143].

In this context, body image disturbance is a key distinctive facet of AN: indeed, a distorted body image representation is a well-known risk and prognostic factor of AN. This key symptom allows one to understand how anorexia patients act according to their distorted perception of the body [32, 56].

First, it is relevant to highlight the interplay as well as the distinction between body image and body schema—two of the many different definitions of body perception discussed in the field of eating disorders [137]. Body schema is a long-term representation which contains fine-grained spatial contents required to perform, plan, and control successful movements. Body image, instead, is a long-term and richer dimension of self-identity. The body image provides important sources of information, not only about the physical body itself and its properties or configurations, but also about the surrounding environment and how the individual interacts with it. The body image representation allows comparative judgments in respect to others and enables perceptual representation of our bodies as objects in the world we perceive [33, 87, 93]. Specifically, body image is determined by how patients believe their body is rather than how it really is, leading to the so-called experience of being “locked to an objectified body” [93, 105].

Body image distortions have always been a major focus of behavioral, physiological, and neuroimaging studies. These studies long for identifying prognostic as well as therapeutic-target neuropsychological markers of this self-identity concept. Most of the research in this field originally implemented perceptual/visual assessment methods in order to evaluate how patients perceive and estimate body shape, size and weight, by equating ‘‘body-image disturbance’’ with a perceptual distortion [34, 37]. Nevertheless, taking into account current studies in this regard, there is a general consensus on the fact that body image should be considered multidimensional in its components. This framework could thus allow us to understand the ways patients perceive, feel, think and behave according to their body [4, 34, 37, 95].

Far from being a unitary concept, body image is characterized by perceptual, affective, and cognitive dimensions. The first component encompasses the visual detection, estimation and identification of the body’s properties and the accuracy of evaluating one's own body proportions (size, weight, shape). The second component enables information about the feelings and emotional regulation strategies developed towards one's own body image (e.g., satisfaction/dissatisfaction feelings, anxiety, interoceptive awareness). The third one outlines beliefs, cognitive representations, attitudes, and biases which influence the evaluation and decision-making processes [37, 98]. Furthermore, there are few studies which have also investigated the behavioral and multisensory counterpart of body image. This framework leads to focus on motor, proprioceptive, somesthetic, and kinaesthetic functions which can be outlined as important properties of body schema. In this regard, somato-sensory/somato-motor facets of body representations are altered and influenced by one’s distorted body image [22, 59].

In this context, it is also important to account for physiological markers which characterize each component of body image (e.g., heart rate, skin conductance, eye gazing). These autonomic markers could give us insights in the relationship between sympathetic/parasympathetic balances beneath distorted body image as a disorder-trait. The findings of these latter studies are often inconsistent due to common physiological blunting issues in AN. Indeed, there is compelling evidence that anorexia nervosa is associated with blunted responsiveness to different kinds of physiological markers, when exposed to salient eating disorder stimuli [12]. Notwithstanding, these approaches have outlined relevant physiological differences between AN patients and healthy controls, mostly perceptive and affective ones, when patients are exposed to their own or other’s body models [38, 52, 103].

To the best of our knowledge, the state of art of body image disturbances in anorexia nervosa reveals several consistent findings as well as limitations. Regarding the most important common findings, patients with AN consistently overestimate their body size, leading this distortion to perceiving others as thinner, and thus extending the pathological facet of this trait to the perception of others body [90, 113]. Higher levels of body dissatisfaction and drive for thinness are described as well-known maintenance factors of the disorder, accounting for cognitive aspects of this disorder-defining trait [37]. Studies suggested also that individuals with AN have an altered body schema, with an impaired ability to integrate visual and proprioceptive feedback about their bodies [42]. Reduced emotional and interoceptive awareness is often accounted for in this regard, displaying reduced awareness of internal bodily sensations (like hunger) and emotional states [94]. As far as neuroimaging studies are concerned, abnormal activation in the occipital and parietal lobes are highlighted, accounting for visual as well as somato-sensory distorted mechanisms of how body representations are shaped in patients. The majority of studies in this field, pointed out the role of the fusiform gyrus, responsible for processing detailed visual information: this area shows hyperactivation when individuals view distorted or oversize versions of their body [123, 131]. Other neuroimaging studies showed consistent patterns of functional activations also in the insula, a region critical for integrating internal body sensations, which is often hypoactive in AN. This dysfunction is associated with poor interoceptive awareness and may contribute to the distorted self-perception of body size, accounting for a more emotional counterpart of body image [63]. Finally, another consistent result is related to the ventral striatum, part of the brain’s reward system, showing reduced activity in response to food stimuli and to viewing one’s own body in individuals with AN. This suggests that the body dysmorphia seen in AN may be linked to a disrupted reward system, where thinness is overly rewarded and normal body sizes are perceived negatively [138]. Of note, focusing on functional connectivity patterns, the Default Mode Network (DMN)—which is involved in self-referential thinking and body awareness—shows abnormal connectivity in individuals with AN. These alterations may contribute to the rumination and anxiety related to one's own body image and weight perception in AN [26, 78].

Nevertheless, several caveats could be pinpointed from these studies. First, it should be mentioned that anorexia nervosa is a highly heterogeneous disorder with variations in severity, clinical subtypes (e.g., restricting type vs. binge-purge type), and duration of illness. This heterogeneity makes it difficult to generalize findings across the population of individuals with AN [139]. Most of the studies, particularly neuroimaging studies, suffer from small sample sizes, which reduces the generalizability of the findings [29]. At the same time, most studies use cross-sectional designs, which do not allow for conclusions about the causality of the observed neural or behavioral changes during time. Incidentally, the effects of malnutrition and starvation lead to the question of how to distinguish whether the findings are due to the eating disorder itself or to the effects of prolonged starvation [5]. One of the main limitations is also the lack of standardization of body image tasks: the tasks and measures used to assess body image distortion—in both behavioral and neuroimaging studies—vary significantly, thus making it difficult to compare results across studies [37, 41]. Of note, it is relevant to use more consistent terminology and more specific definitions of body image constructs as well as experimental designs, powered samples, and well-validated measures [40]. Incidentally, most of the studies focus on individuals in an acute state, with less emphasis on those in the weight-restoration stage [24]. Finally, to the best of our knowledge, poor evidences are present in literature regarding the role of the sensorimotor dimension of body image construct—which support the interplay between body image and body schema—as well as physiological markers of this disorder defining trait [22, 24, 40, 52, 59].

Therefore, as most of the studies are underpowered and involve a possibly heterogeneous pool of clinical and experimental factors, literature reviews are needed to synthesize their results in order to identify consistencies and differences. Starting from these common findings and gaps, our article aims to review body image studies in AN which have implemented both behavioral and neuroimaging methods, without focusing only on a specific body image quantitative assessment (either psychometric or brain measurement). This could allow us to give an overall and timely background of the state of art of body image behavioral mechanisms, together with a discussion on the specific brain mechanisms which support the neuropsychological disturbances associated with body image distortions. At the same time, we also focused our attention on both the somato-sensory/somato-motor component and physiological markers of body image disturbances, which are not widely deepened by current studies so far, and yet insightful dimensions of this self-identity construct [52, 59]. Thus, we classified each study according to the different and shared degree of involvement of each body image dimensions (perceptual, cognitive, emotional and somato-motor/somato-sensory), pinpointing specific functional brain correlates and physiological markers which integrate the common findings about this disorder-defining trait. The research questions which lead this scoping review can be summarized in what we can learn from behavioral, neuroimaging and physiological studies that have focused uniquely on AN, regarding body image as a multidimensional construct. And mostly, starting from common findings and limitations of these studies, what could be further accomplished to understand body image as a key prognostic and maintenance disorder-defining trait of AN.

## Method

### Search strategy and eligibility criteria

We used the following keywords: (“Anorexia nervosa”) AND (body image). All keywords were entered in three databases: Pubmed, PsycInfo and Scopus. All articles that have been published after the date of 30.04.2024 are not included. We have searched for body image taking into account all the studies which have explored its different core dimensions (perception, emotion, and cognition, somato-sensory/somato-motor). The primary aim was to dive into the multidimensionality of it, without focusing only on a specific facet.

The titles and abstracts of each resulting hit were examined. We first established four main eligibility criteria: (1) original peer-reviewed research articles, in English language; (2) anorexia nervosa diagnosis as the main clinical group focus, and no other groups with eating disorder according to established guidelines (e.g., DSM/ICD); (3) comparison with healthy controls; (4) quantitative assessment, with behavioral, physiological and/or brain measurements. Articles not meeting these criteria were excluded. We thus tried to evaluate the first three criteria by screening the abstracts: articles that met these criteria or in case one or more could not be evaluated based on the abstract, were screened in their full text. The last criterion was assessed and the others were validated based on the full length article.

**Fig. 1:**
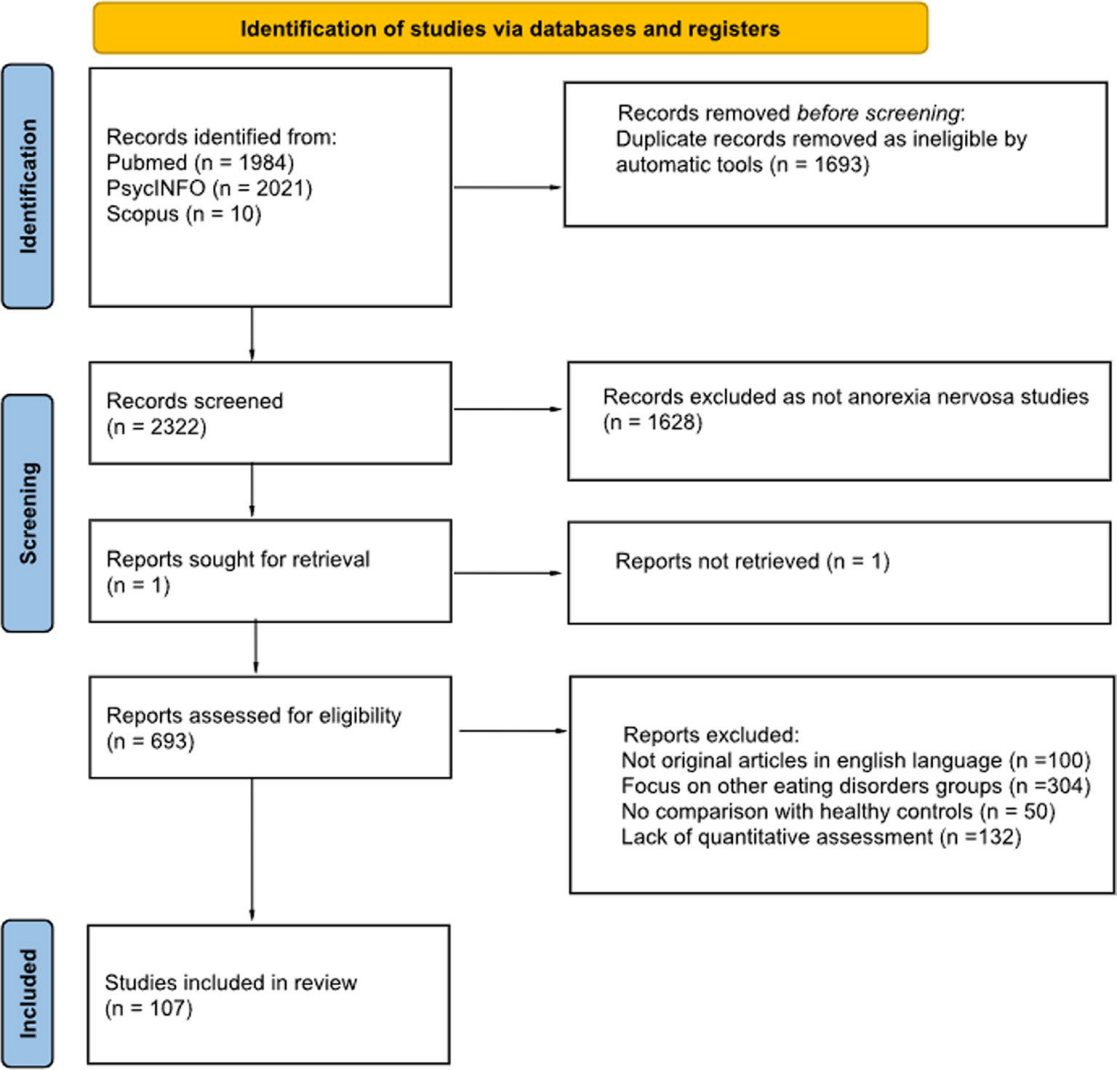
PRISMA flow diagram illustrating the study selection process, including the number of records identified, screened, excluded, and included in the final review

We did not consider confounding factors such as the sample inhomogeneity (e.g., AN subtypes), the presence of psychiatric comorbidity and the pharmacological history. The search strategy and the process are schematized by a PRISMA flow chart (see Fig. 1).

### Taxonomy criteria

We have first categorized the studies in two main clusters based on the methodological design used (behavioral studies or neuroimaging ones). Second, we classified the retained studies using the same taxonomy method exploited by Gaudio and Quattrocchi [37]. Following this methodology, specifically, we have started our classification based on the assumption that body image is multidimensional in its facets. Therefore, we have considered studies that have explored the perceptual, emotional, and cognitive counterpart of this identity construct. As far as the classification method is concerned, we have then have applied the degree of involvement of each dimension of body image distortion as primary (predominantly involved), secondary (involved to some extent, but not predominant) and no involvement (not present at all), both for the behavioral, physiological and neuroimaging studies. This methodological framework has allowed us to shed light on all the dimensions. We have also accounted for the fact that, for a part of the studies reviewed, there is a simultaneous presence—to some extent—of each component, which cannot be disentangled from one another when discussing each dimension's role in shaping body image experience.

Nevertheless, we conducted a further step. Besides the cognitive, emotional, and perceptual counterpart, we have also included somato-sensory and sensorimotor research regarding body image, given the importance of how kinaesthetic, proprioception, and motor functions rise by the interplay between body image and body schema [19, 22, 76]. Plus, we have also retained studies which have deepened physiological markers (e.g., eye gazing, heart rate, skin conductance response) outlining each component.

## Results

Our database search on body image studies in AN yielded a total of 4015 articles. Based on the eligibility criteria, the papers screened and included in the final review were 107 behavioral (84) and neuroimaging (23) studies (see Fig. 1). Of these 107 overall studies, only one study can be classified as cross-sectional and longitudinal design, three as longitudinal and the remaining 103 articles as cross-sectional.

Of note, of the studies which have explicitly specified the subtype criteria: 16 studies have focused on restrictive subtype (AN-r); two on binge-eating/purging subtype (AN-b/p); 19 studies on both AN-r and AN-b/p; four on atypical subtype; 11 uniquely on weight- recovered state (WR). Furthermore, three of these studies included also male patients. The mean age across all the studies is 20.1 years (3.79 years SD).

Table 1 reports the paradigm, sample characteristics, aims and main findings of behavioral studies (cross-sectional and longitudinal). Table 2 reports the paradigm, sample characteristics, aims and main findings of neuroimaging studies (cross-sectional and longitudinal).

Table 3, reports the classification criteria—according to the degree of involvement of each component—for behavioral (label A) and neuroimaging studies (label B)—accounting for the classification method mentioned above. The degree of involvement is estimated depending on the methodological paradigm, key purposes, and dimensions explored in each study. Each approach is also labeled in order to recognize the investigation of possible physiological markers of body image (label C).

### Perceptual component

The most reliable finding of studies (n = 14) which focused their attention on the perceptual counterpart of body image, is the pattern of overestimation of different body measures (size, weight and shape) and of body salient dimensions in AN [13, 39, 72, 86, 91, 102, 106, 114, 121]. Incidentally, AN patients show a detailed-based visual information processing, during the assessment of body visual stimuli: this means that visual information processing of body stimuli is guided by a biased visual attention on specific body features rather than on the global representation of the whole body [134]. Along with these perceptual disturbances, physiological studies

(n = 7) confirmed an altered pattern of eye gazing fixations, which turn out to be wider as well as an altered visual scanning behavior encompassing specific body parts, persisting in weight-restored stage [16, 136].

Common cortical-subcortical circuits can be highlighted during their own body image representation in AN. First, according to literature already widely accepted findings, one study here reviewed showed an increased activation of the left extrastriate/left fusiform body area (EBA-FBA), involved in the visual processing of body and face perception [24, 123, 124]. Along with these results, an overall increased functional activation of the limbic system (e.g., insula) is outlined, as perceptual-affective reactivity to body stimuli, which appear to be emotionally salient for patients [111]. Frontal-parietal circuit is characterized by an increased functional activation in AN, sustaining attentional as well as perceptual biases in the representations of body stimuli [135, 137]. This latter finding goes along with the salience of DMN principal nodes functional activation, specifically during own body evaluations tasks, suggesting the specificity of DMN as potential candidate for distorted own body image representation in AN [135]. An altered integration between ventral striatal circuit—reward system—and frontal striatal circuit is often discussed in this studies category, strengthening distorted reward processes as responsible for altered visual estimation mechanisms of body stimuli [128]. This distorted reward system supports a persisting bias towards viewing lower weight individuals as more attractive. Moreover, an activation of the medial frontal gyrus during weight restoration state has been demonstrated to be mostly active as a cognitive compensation mechanism of disorder body image disturbances during appraisal (evaluation) mechanisms of the self, uniquely in AN [128]. Finally, an increased activation of the medial parietal gyrus and connectivity of the somato-sensory nodes (parietal network) have been also demonstrated in AN, during other’s body image stimuli processing, due to high level of social comparison with other body models prototypes [80, 108, 111, 124, 128, 135].

Controversial and mixed results, however, could be drawn by these studies. First, it is not clear if AN patients process their own and other’s body stimuli in the same way, due to the heterogeneity of tasks design to assess body image evaluation and estimation processes [71, 79, 80, 128, 137]. Second, physiologically speaking, depending on the type of paradigm, pattern of hyper/hypo-visual scanning behaviors are described, along with different duration of time fixations and attentional biases towards body salient dimensions [45, 136]. At the same time, changes in physiological markers are not always straightforward: for instance, in one study here reviewed regarding pupil reflex response—in which anorexia nervosa show higher pupillary response to own body stimuli—turns out to be not exclusive to clinical samples and not linked uniquely to a perceptual counterpart of body image [17]. Third, depending on the type of methodological approach used (e.g., distorted versus undistorted body visual stimuli, mirror exposure), an hyperactivation or hypoactivation of the frontal-limbic system can be found [108, 111]. Moreover, there is no clear weight restoration conceptualization (time of being outpatients as well as a very small number of studies focused on WR state, not allowing a generalization of the findings according to a post-acute state of patients. The design of these studies is predominantly cross-sectional. At the end, other two important factors should be considered: first, a special focus on the young adulthood stage can be spotlighted, with only few studies on other developmental stages,second, the majority of the studies regarding the perceptual component either are focused on restrictive subtype or either did not specify the subtype of the disorder, with an underrepresentation of the other kind of AN subtypes.

### Cognitive component

In comparison to perceptual findings, cognitive approaches yielded more consistent and convergent results regarding the global role of attitudes, cognitive beliefs, and thoughts behind the concept of body image. The majority of these studies (n = 6) are methodologically characterized by the use of body related words or adjectives, leading to the overall following findings in AN [9, 49, 129, 140]. First, memory biases/altered recall autobiographical memory systems during body-related retrieval tasks, together with attentional biases, and altered executive functions (cognitive interferences) when patients are confronted with body-word stimuli. A strong drive for thinness and weight stigmatization are well represented by these studies as cognitive maintenance factors influencing the representation of one's own body in AN (n = 7), during these kind of cognitive tasks: these findings go along with an impaired embodied body image cognitive schema, which can be reflected in the ways representations of the own body influence also body schema dimensions [9, 49, 53, 129, 140]. Implicit, automatic and distorted cognitive biases elicited by body category stimuli have also been found in some studies (n = 6), in this regard [8, 10, 31, 49, 74, 129]. Of note, in two studies it has been proved that thin-body BMI models influence the learning and decision-making processes which characterize restrictive behaviors, accounting for risk factors which endure also in WR state [54, 66].

Regarding the neuroimaging studies (n = 3), an hyperactivation of the somato-sensory cortex has been demonstrated, supporting a distorted autobiographical memory as well as emotional suppression when AN patients are confronted with body related words [129]. Hypoactivation of angular gyrus, precuneus and posterior cingulate cortex go along with these findings [129]. At the same time, the left hemisphere appears to be dominant in supporting an altered interoception awareness interplay with cognitive beliefs around body image representations [58, 117]. As far as social self-identity appraisal processes are concerned, an impaired activation of precuneus/ventral anterior cingulate cortex and prefrontal cortex have been showed, with a stronger activation of the medial frontal gyrus as well as dorsal anterior cingulate cortex to cognitively compensate distorted social self-identity evaluations. At the same time, social cognition network and Salience Network seed regions have been suggested as potential biological markers of AN, as supporting distorted cognitive and social appraisal processes of the self perception in AN, perduring in WR stages [71, 144].

One limitation of these studies, to the best of our knowledge, is that there is scarce evidence and investigation as regards the neuronal counterparts which sustain these cognitive distortions behind body image beliefs and representations; at the same time, at the best of our knowledge, no evidence has been found in regards to the physiological markers of these cognitive impairments. As for the perceptual studies, the cross-sectional nature of these study designs is another important limitation. No clear weight restoration conceptualization (time of being outpatients) as well as very small number of studies on this clinical state, remains as another controversy. Finally, a special focus is dedicated to the early-adulthood stage, with scarce evidence of other developmental stages; at the same time, there is a overall underestimation of all AN subtypes (restrictive, binge-eating/purging) as the majority of the studies found for this component did not specify the AN subtype profile: this final gap could not allow us to generalize results to other kind of AN subtypes.

### Emotional component

The majority of the studies found in this body image category are longing for finding affective physiological markers of body image in AN (n = 5), as well as insightful and consistent behavioral/neuroimaging sustaining factors of body image disturbances (n = 12). In summary, from physiological studies which implement skin conductance response (SCR), startle reflex and heart rate (HR) approaches, it has been concluded that there is some degree of dissociation between psychophysiological reactivity and subjective response to own body exposure [64, 70]. In other words, from a physiological point of view, it is difficult to disentangle and outline what comes first in the relationship between the sympathetic and parasympathetic affective response with cognitive control processes. These findings could be reflected in the fact that, even though patients tend to subjectively report an affective/emotional trigger when confronted with body stimuli, physiological levels during these kind of tasks could be blunted [64, 70]. Nevertheless, it can be denoted an emotional salience evaluation which influences body image representation, supported by the presence of attentional emotional bias toward body images and by altered interoceptive awareness [52]. Anxiety seems to support emotional and cognitive body image disturbance symptoms: accordingly, AN have less activation in the medial prefrontal cortex and right fusiform gyrus in response to body checking stimuli [125]. Interestingly, anxiety seems to support attentional biases which are the foundation of the drive for thinness and distorted perception of body image stimuli [100]. There is an inability to regulate negative affect in response to body images via pregenual anterior cingulate cortex activation during body comparisons, which persist after recovery [65]. Recovery may involve the restoration of a successful top-down control of negative emotions triggered during self-other comparison of body images, at the expense of activating compensatory functions in the pregenual area in order to face negative emotions. In addition, implicit affect toward pleasant and unpleasant stimuli may be potential markers of acute AN, dissipating with recovery (Spring et al., 2014).

Compensatory neural mechanisms that prevent emotional responses from disturbing cognition relies also on the prefrontal cortex [99]. Higher affective value is given to oversize BMI body models, with an increased activation of the dorsal prefrontal cortex [14, 18]. Dysfunction within the frontal-insula-limbic-striatal circuit has been discussed in the context of failure to regulate inputs from higher level cognitive representations about the importance of weight and shape [85]. Through an evoked-responses potentials paradigm, it has been shown a pronounced emotion intensity level in response to underweight body stimuli in AN: these affective reactions stand for a greater capture of attention and deeper processing of body stimuli with a specific BMI type [101].

The most controversial results regarding this body image component are related to the physiological studies, due to physiological blunting issues which don’t allow us to differentiate between objective emotional markers and subjective appraisal/evaluation processes of patients [64, 70]. Another limitation is the cross-sectional nature of these designs as well as the special focus on the early-adulthood and acute state, with only few studies focused on the effects of emotional coping and regulation strategies—in combination to cognitive compensation processes—in the WR stage. At the same time, restrictive subtype is overrepresented in comparison to other AN clinical subtypes for the studies found regarding this specific body image component, highlighting the impossibility of generalizing the main results of this component to other clinical profiles.

### Somato-sensory/somato-motor component: body image and body schema

Impairments of the overall network involved in the emergence of body schema and body image in AN’s own perspective judgments have been highlighted for this particular body image component. The majority of the studies in this regard (n = 14) point out how body image representations disturbance in AN could be considered as pervasive in the ways these representations affect not only (conscious) cognition and perception, but also (unconscious) action as well [59–61, 145].

Not only actions, but also somato-sensory aspects of body image are considered impaired: body image disturbances in the tactile and visual modality result from top-down influences of body dissatisfaction on the mental body representations, which are necessary in tactile size estimation and visual imagery [59]. Overall, they do not limit themselves to body dissatisfaction and unrealistic visual mental images of the body but extend to deficiencies in somato-sensory perception [59]. Body size and body shape, both aspects of body image, result also from the integration of visual and proprioceptive information. Patients perceive things differently as a result of body representations and beliefs concerning body size, which influence the specific somato-sensory process of tactile experience [59, 61]).

Nevertheless, body representations are susceptible to plastic changes and that this might be an important purpose for treatment [61, 145]. Body representations are constructed from and reciprocally influenced by touch stimulations, basic somato-sensory mechanisms of AN are not impaired [112]. For instance, it has been shown that touch processing in AN is not related to an aberrant elementary tactile mechanism, but to a more complex social, affective and maladaptive mechanism related to brain reward and body image systems [19]. No primary tactile cortices functional activation differences have been found between AN and healthy controls (HCs). Yet, differences have been found in networks including left caudate nucleus as well as bilateral occipital cortex, showing both an affective and perceptual body image—body schema disturbances [19]. Other studies (n = 4) have shed light on the fact that there is an altered integration of multisensory bodily inputs, exteroceptive and interoceptive ones [19, 61, 112, 145].

One limitation of these kinds of studies is, to the best of our knowledge, the lack of physiological and neuronal depth analysis of how body image and body schema interact. As for other studies mentioned regarding the other body image components, a special focus is given to early-adulthood, to implementing a cross-sectional design and to not deeply studying the WR state. At the end, as for the cognitive component, there are not sufficient studies to well represent all the AN clinical subtypes, leading to difficulties of finding generalization.

## Discussion

Body image is a key prognostic and endophenotypic factor characterizing AN patient's experience of their own body [34]. Indeed, the body image influences the ways patients perceive, emotionally regulate and cognitive process the environment around them, according to their distorted perception of the body as an “object” [93]. Body image distortion is multidimensional in its facets [4, 37]. We here discuss the main results for each component role in shaping the experience of body image, underlying a link between psychological processes, cortical-subcortical networks patterns and bio-signal markers. Of note, we have discovered that for some studies—mostly the recent ones—it is difficult to disentangle the contribution of each component from one another. Therefore, some of the findings discussed could be the result of a combination of two or more components, with a peculiar emphasis on the ones which were of primary importance of investigation for each study (see Table 3). Of note, the majority of the studies here discussed are focused on restrictive subtype of anorexia nervosa, in acute state and in both adolescence/adult stage: scarce evidences, at the best of our literature screening, have been found to well represent how body image is shaped in other type of AN subtypes, in WR state as well as in children developmental stage. Thus, more literature reviews are needed to address the heterogeneity issues of AN findings regarding body image construct.

### Body image as not “pure” perceptual disturbance

Throughout the investigation of body measurable components (body size, body shape and body weight perception), studies mostly focused on the perceptual component of body image have proved a perceptual tendency to overestimate the width of the own body in AN [13, 27, 68, 72, 83, 88, 91, 114, 121, 141]. These studies (n = 14) primarily relied on visual size estimation approaches, image marking procedures as well as other size estimation techniques. Weight restoration (follow-up in a variable period between 26 to 213 days) has been proved to lessen the pattern of overestimation of body width in AN, assumed as a relapse factor [106, 114]. Furthermore, a tendency of overestimating perceptually salient body dimensions (e.g., abdominal and pelvic areas) can be highlighted [39, 102]. Moreover, in one study it has been underlined how physical exercise seems to contribute and exacerbate body image distortion experience [21]. Only three less recent studies have discovered a trend in underestimating—and not overestimating—own body shape, but with the implementation of distortion/morphing techniques different from the utilization of image marking stimuli [35, 72, 130]. Indeed, these latter studies showed mixed results, only partially confirming the overestimation pattern.

Nevertheless, according to one study here reviewed and supported by other behavioral/neuroimaging studies, a direct confrontation with the appearance of the body (e.g., mirror gazing tests) has been demonstrated to be a more sensitive measure of perception and feelings about the somatic appearance [36], Pierloot et al., 1978; [20]. The anxiety trigger posed by a mirror confrontation leads to a higher activation of amygdala, together with right fusiform gyrus and brainstem regions, caused by aversive, threat-related events and by a potential recall of aversive memories [111]. The “fear network” (limbic system) might be important to support and maintain body image biases, going beyond the perception component [111]. Incidentally, cognitive, perceptual, and emotional functional systems could be possibly suppressed during body image stimuli presentation, in contrast with previous findings related to the “fear network” [111]. Nevertheless, this finding could be related to the presentation of undistorted images and so the differential impact of distorted/undistorted body pictures to the limbic system activation [108].

Morphing techniques of own and other body images (body photographs, videos, and computer- based stimuli with a specific degree of BMI scale) have been also greatly exploited, mostly by current studies (n = 22). In acute and after treatment stages, the pattern of overestimation is shown to affect not only the perceptual estimation of the body stimulus (e.g., mirror condition), but also memory (estimation of body size according to one’s memory and cognitive beliefs). Incidentally, stabilized perception and expectations regarding one's own body weight play a role in this context [86, 132].

Even though it appears that overestimation of one's own body representation is a consistent processing pattern found in AN, the same cannot be said regarding other’s body representation processing. According to two studies, functional activation changes in the frontal-parietal network (e.g., inferior parietal sulcus) as well as—in a functional connectivity perspective—in the DMN, have been interpreted as important sustaining factors for body image disturbances [135, 137]. Specifically, disturbances in the frontal-parietal network has been interpreted as a biological expression of a disturbance in the visuo-spatial processing of the own body shape [137]. Incidentally, AN patients show a hyperactivation of the dorsal posterior cingulate cortex during their own-body processing, but a response failure to another's body processing at the precuneus and ventral posterior cingulate cortex locations. These results come along with a decreased resting-state connectivity between the dorsal posterior cingulate and the angular gyrus. Overall, these nodes are part of the DMN and can be representative of self-directed impairments during body image perception evaluations [135].

Other studies (n = 2), in the opposite direction, pinpointed an increase of activation in signal in different frontal-parietal areas during the exposure to other’s body stimuli models: specifically, an increasing of functional activation of the medial prefrontal gyrus only in AN has been revealed, for intense social comparisons with other women’s body models [108]. An impaired connectivity pattern in the somato-sensory components of the parietal network, while viewing others’ bodies in AN, has been also discovered [80]. In this direction, shared similarities between right parietal patients and anorexic patients, during the estimation of body’s boundaries, can be outlined [84]. These findings shed light on a perceptual and emotional trigger arising from other body exposure at some extent [79, 108, 115].

Another consistent result deeply investigated in literature during the exposition to body visual stimuli is the activation of the fusiform body area as well as the extrastriate body area, and the specific information processing of visual body stimuli in AN. The effective connectivity from the left FBA into the left extrastriate EBA is characterized by a significant negative correlation with the body size misjudgment scores, suggesting alterations of finer-grained perceptual analyses of body shapes [24, 123, 124]. Furthermore, activation patterns are largely different from other mental conditions characterized by disturbances of body image features, like for body dysmorphic disorder [133, 134]. AN patients seem to have a paradoxical advantage of visual discrimination of body morphology, which is a detail-based processing of the body (despite a configural process). Yet, this means that this information processing “advantage” leads to attentional biases and obsessive worries during the evaluation of body image, which do not arise from a simple visual deficit [133, 134].

Eye gazing paradigm studies (n = 7) confirm the previous less recent and current studies common result regarding the altered pattern of overestimation of body size and of body salient dimensions. Anorexic observers find bodies with lower BMI more attractive, with wider fixation patterns encompassing specific body parts. AN “avoid” looking at these areas of their body of which they have a particularly negative perception [136]. Contrary to this finding, another study suggests that the attentional bias to one's own subjectively unattractive body parts might represent a mechanism maintaining body image disturbance in women in general, with longer fixation patterns on the subjectively unattractive body parts [45]. Indeed, this latter trait endures also in weight recovered state, and it is independent from accuracy in body size estimation [16, 38, 136]. In this context, the presence of persistent attentional biases comes along with a hyper-scanning behavior (increased fixations of shorter duration) regarding the identification of body size [89]. More visual scanning at thin body shapes characterizes the visual scanning behavior of AN in a hierarchical fashion, rather than social interactions and similarly to overweight body shapes [67, 92]. Furthermore, AN patients show higher pupil response reflex towards their own bodies: this allows us to take into account important attentional bias to self-related salient information. Nevertheless, in a controversial way, it has been also found that pupil response to underweight stimuli differed from pupil response to normal/overweight stimuli in both AN and HCs groups. Weight gain was associated with an increase in pupil response reflex, an improvement of ideal BMI and a decrease of body dissatisfaction, but no change in perceptual distortion [17].

The implementation of all these procedures has led to discover another common perceptual- cognitive pattern in AN (n = 10): the explicit drive for thinness [3, 50]. A significantly higher ideal body perception index in AN patients, is strongly directed to underweight bodies [47, 96]. This pattern might come along with a highest ventral striatal activity [28]. The brief exposure to round models can increase and emphasize that there is an esthetic and perceptual sensitivity strongly biased for thin models, across different patients’ age [15, 73, 116]. Only in the AN group, indeed, there were observed activations in the posterior cingulate cortex and parietal cortex, as well as a hypoactivation of prefrontal and insular areas for underweight images [55]. Finally, it has been underlined a pathological integration and regulation of the frontal striatal circuit and limbic system [55, 128]. Notably, the more sustained the disorder course, the greater the decrease in activation in the reward system subcortical structures, in the caudate and in the nucleus accumbens (social proficiency). The increased recruitment of medial frontal gyrus in AN-WR may have reflected the need to actively employ cognitive reappraisal strategies when considering the self, a pattern not seen in actively disordered individuals and not needed in healthy controls [128].

As far as sex differences are concerned, only one study focused on the fact that female patients are more influenced by specific body image categories like weight and shape. These sex difference disorder-specific traits seem also to endure in WR state [110].

In conclusion, from the implementation of cutting-edge methods like virtual reality ones, it has been pinpointed that the attentional and perceptual biases behind body image could be lessened (Porras et al., 2020). Regardless the pattern of overestimation of own body image and body dimensions, from these current perspectives (n = 5) it is stressed that body size overestimation is associated not with a perceptual disturbance, but rather with the severity of cognitive (drive for thinness) and affective (body dissatisfaction) features [44, 69, 81, 82, 97, 104]. A recent study testing the visual misperception hypothesis (body image as a pure perceptual deficit) indicates this important issue. Using a body size estimation paradigm, it was shown that it is true that AN patients overestimate their body size, but these results don´t confirm that overestimation stems exclusively from a visual distortion and response bias effect towards their own body. Finally, we can conclude that other non-perceptual factors should be considered [34].

### Body image representations and beliefs

The majority of these studies have been focused on presenting anorexia-related words and/or sentences about the experience of body image (e.g., body weight, body shape, food), in order to evaluate different cognitive processes (n = 6). Patients showed a strong explicit memory bias for anorexia related words [9, 49, 140]. Information related to weight, shape, and food is well embedded within the memory structures of patients, with a strong conscious rumination and elaboration of these kinds of words. Indeed, when AN patients have to recall autobiographical memories in response to both AN-relevant (e.g., food/body-related) as well as neutral cue-words, overgeneralized memories for eating disorder cues could be highlighted. This leads to a stronger functional activation of the somato-sensory cortex in AN versus precuneus and angular gyrus for HCs, supporting a distorted autobiographical memory as well as emotional suppression when AN patients are confronted with body related words [129].

Another widely less recent and still current approach is the Stroop-revised task: it allows to explore attentional biases and executive functions towards eating disorder related words [25, 109]. Body image words related to the two extreme poles of BMI (thinness and fatness words) were shown to be salient for AN patients, with no preconscious attentional biases but rather an explicit attentional distorted processing of these stimuli. Furthermore, a delayed color naming among women with anorexia for both body shape categories is visible, according with the varying emphases on the drive for thinness [8, 11, 53, 107, 109]. Patients are conditioned by the meaning of the stimulus words in the “weight/shape and lightness and agility'' category, with the least number of errors in these categories [25]. The role of attentional biases is evident when AN patients are confronted with specific body image categories (e.g., underweight prototypes), which can be accounted for disorder-specific information processing [100]. This finding is important, when one considers attentional biases as a possible promising candidate for maintenance factors of the disorder [100].

A significant cognitive and emotional weight stigmatization in AN could be also highlighted from these studies (n = 7), together with an impaired embodied body image cognition [7, 10, 74, 118]. These studies allow us to stress how these patients function under unrealistic cognitive representations of their own body, which maintain the disorder [57, 58]. Together with cognitive beliefs, an important role for explaining body distortions is given to low levels of interoception awareness, which affect the way patients judge their own body image rather than the other body models [117].

Another novel methodological approach can be found in another study here reviewed, confirming the role of attentional processes and top-down cognitive control in AN. Fusco and colleagues (2023), tried to probe the link between activation of body representations and cognitive control throughout a Flanker Task [23]. Overall, when the overweight body depictions were presented as flankers, these were likely perceived as threatening stimuli by AN patients. Consequently, they are able to capture attentional resources, thereby weakening the processing of targets taking the form of underweight bodies and increasing cognitive interference [31]. Specifically, the AN group tended to avoid categorizing a high-calorie food for a low-calorie food, and to avoid categorizing an overweight body for an underweight body [66]. This finding is particularly important when one considers how AN are characterized by a distinctive cognitive mechanism which drives them to avoid certain types of errors when categorizing food and body stimuli. This result highlights a high-risk perception and cognitive mechanisms which sustain body image disturbances [66].

In the end, only two studies reviewed here have explored a very different cognitive counterpart of body image, by focusing on appraisal processes. These studies have implemented a Social and Physical Identity Appraisal Tasks, studying the neural circuitry involved in one’s identity in weight recovered state [71, 144]. There are strong impairments in precuneus and ventral anterior cingulate cortex during social identity evaluations, with activation of other regions (medial frontal gyrus/dorsal anterior cingulate cortex) to compensate for these deficits. These impairments are only evident in the self-condition, with no differences regarding physical appraisal evaluations between self and others. Overall, these data suggest an impairment in one of accurately evaluating one’s identity, socially speaking, in AN. Nevertheless, one of the most important findings are the strong differences in medial prefrontal cortex and seed salience network regions (such also precuneus), as well as in social cognition networks in AN-WR. These regions could be accounted as biological traits of the disorder. Together, an overall significant portrait of how identity—whose body image and body schema are core dimensions—is experienced by AN patients could be drawn from these studies [144].

In conclusion, we have also reviewed one novel study regarding how the cognitive—as well as social—counterpart of body appearance influences reward and decision-making mechanisms [54]. The unique findings of this one study are related to the fact body image appearance can have a significant effect on risk behaviors attitudes. In particular, thin body models drive AN patient (both in acute and WR state) to an aversion to risk (rather than loss), with desirable body outcomes (body appearance) and undesirable body outcomes linked to greater risk aversion. This finding shed light on how restrictive behaviors are the result of dysfunctional learning and decision processes: this enduring trait, of note, can be accounted for as a risk factor in healthy controls too [54].

### Interoceptive awareness and autonomic affective reactivity to body image stimuli

Most physiological studies investigating different autonomic markers in AN in relation to body stimuli, are here reviewed (n = 5). Knejzlíková and colleagues (2021) recorded skin conductance during rest, neutral and mirror stimulus exposure tasks. Skin conductance responses to either stimuli were not different between AN and HCs, although patients had a significantly lower baseline level of skin conductance. The increase of SCR during the mirror exposure compared to neutral stimuli was higher in patients with stronger body dissatisfaction and interoceptive sensitivity [64]. As far as startle response effect is concerned, both acute and weight-recovered AN patients and HCs show an inhibition of startle response to eating disorder-related salient categories, like shape/size of body stimuli [70]. Nevertheless, acute state and WR showed startle suppression to high-calorie foods as well as normal-weight body images. Interestingly, AN groups show enhanced levels of attentional biases to eating disorder-related stimuli, due to excessive state of anxiety towards feared stimuli like body images [70].

The highest HR decelerations in response to pictures of strongly underweight and overweight women have been also explored. These findings may reflect emotional processes such as anxiety due to social comparison, and these effects are present in both groups. Notwithstanding, an overall greater HR change towards all bodies categories as well as stronger HR reactions—decelerations—were found to body stimuli, denoting an emotional salience evaluation behind body image for AN [52]. On the other hand, the combination of multiple biomarkers (startle reflex, SCS HR) showed a significant startle inhibition in AN for emaciated bodies, with scarce evidence of differences in HR parameters [103]. The eye gazing patterns in AN, as described in one eye tracking study here reviewed, were characterized by a gaze frequency and duration towards the ugliest body part that was significantly higher than in HCs [127, 142]. Although controversy, the overall physiological studies pinpoint then how the exposure to body related stimuli, body salient parts and specific BMI body categories, are able to trigger a significant emotional reaction which is visible by recording bio-signals like SCR, HR, eye gazing, startle reflex.

More consistent and convergent results can be found regarding the behavioral and neuroimaging studies (n = 12). AN reported higher anxiety compared to HCs, positively correlated with body shape concern scores [125]. AN had less functional activation in the medial prefrontal cortex and right fusiform gyrus compared to HCs in response to body checking, and relative to neutral action images. Body shape concern scores correlated negatively with medial prefrontal cortex activation in AN group, suggesting impairments in the self-reference, body perception and social cognition network or in the SN for action/intention [125]. Patients with AN exhibited also significantly greater activation in the pregenual anterior cingulate cortex when comparing their own bodies with images of underweight female bodies. The inability to regulate negative affect in response to body underweight images via pregenual activation, persist after recovery [65, 120].

Incidentally, greater anxiety has been shown during self-other body shapes comparisons, with less body satisfactions rates: this kind of comparison induce more activation of the right sensorimotor brain regions (insula, premotor cortex) and less of the rostral anterior cingulate cortex. This allows us to stress the critical role of insula hyperactivation and anterior cingulate hypoactivation for altered thin models drivenness [18, 30]. In this regard, anxiety can be accounted as an important predictor for significant increase of attentional biases when AN patients are confronted with different BMI body image stimuli [100]. Interestingly, when comparing anxiety with other important clinical parameters (BMI, depression level, eating disorder symptoms), only the impact of anxiety explains why AN patients suffer from attentional biases directed to underweight body image prototypes [100].

Incidentally, dorsolateral prefrontal cortex increased activity in response to the oversize body stimuli, and the direct relation between its activity and shape concern, may represent the need for an increased top-down cognitive control in AN to face emotionally salient cues, such as distorted own image, which may otherwise be experienced as overwhelming and aversive [14]. Other important seed regions are related to a compensation mechanism by a higher recruitment of the insula can be found, during the comparison of the ideal self-body shape with the ‘close to ideal’ thin body images. Incidentally, precuneus activity allows to differentiate between the thinner, actual, and fatter self-images, modulation which is absent in the AN [77]. Dysfunction within the frontal-insula-limbic-striatal circuit has been discussed in the context of failure to regulate inputs from higher level cognitive representations about the importance of weight and shape [51, 85].

When AN are exposed to real and distorted body image by implementing a qEEG system (n = 2), the desired body image has been suggested to be related to right hemisphere dominance. The combination of EEG and MEG systems during a body size estimation task led to the following result: AN patients reported pronounced body dissatisfaction and overestimated their own body size more than controls in the self-referential body estimation tasks. It is interesting to see a mid-to late latency group-specific effect in EEG studies, with stronger linear and quadratic trends and (numerically) relatively higher neural activity in response to underweight and relatively lower neural activity in response to higher weight body pictures in AN. In this view, this effect started mid-latency and involved enhanced (not reduced) neural activity in widespread brain regions argues against a disturbance specific to the neural network of visual body processing in AN [101]. In this context, steady state visual evoked potentials (SSVEP) have been shown to be signatures of important functional processes in AN. These signatures represent synchronized early neural perceptual networks, where increased SSVEP amplitudes indicate activation of larger neural networks compared to decreased SSVEP amplitudes. By implementing a body morphed task, a significantly increase in SSVEPs have been found, when AN patients are confronted with overweight body morphed stimuli: this electrophysiological signature outline an increase sense of fear or disgust (e.g., cognitive-affective component of body image), explicitly. On the other hand, implicitly, the processing of this morphed body stimuli occurs in an automated manner with fewer cognitive resources needed [48].

Finally, focusing on WR state based on one study here reviewed, frontal lobe modulation of emotional regulation is relevant [99]. Women who recovered from AN had more negative affect, higher anxiety, and worse body image than HCs. They also categorized normal weight bodies as more negative and were faster to make that categorization than controls. A greater activation in amygdala, fusiform and medial prefrontal cortex was shown. Overall, normalization of lateral prefrontal function to suppress negative emotions surrounding body image may occur with recovery but other aspects of the network also play a role. The medial prefrontal cortex plays a role in monitoring self-referential emotional responses and controlling them so that other cognitive processes can continue [99].

### Body image and body schema interplay

One of the main research questions of these kinds of studies (n = 14) is if body image disturbances are supported by bottom-up or top-down neurocognitive processes in its relationship with body schema. In this context, throughout the implementation of different body schema tasks (proprioception test, a Finger localization test, right-left orientation test), significant differences occurred only prior to treatment, and only on those tasks most likely to involve functions outside the domain of body schema. These findings suggest a state-related deficit in executive function rather than a specific and enduring deficit in body schema per se [22].

Nevertheless, some of these less recent and current studies (n = 5) have mostly used ecological paradigms in which the participant was not confronted with an external body image per se, but it was required to represent her body’s dimensions in a simulated action, throughout task consisted in making passability judgments (mental simulation) for apertures of different widths [6, 42, 43, 60]. Indeed, a consistent finding across all these studies is the abnormally high 'passability ratio' in first perspective point of view, which is the critical aperture size to shoulder width ratio. This result suggests impairments of the overall network involved in the emergence of body schema and body image in AN’s own perspective judgments. Incidentally, lower accuracy in motor imagery, selective impairment in the mental rotation of human figures, and reduced ability in assuming a different egocentric visuospatial perspective can be added to these results. These findings outline specific alteration in motor imagery in patients with anorexia nervosa and interestingly, patients' difficulties appear to be limited to those tasks which specifically rely on the body schema, which is negatively influenced by own distorted body image representation [75].

Keizer and colleagues have implemented two types of tasks in this direction: Tactile Estimation Task and Distance Comparison Task. The findings suggest that AN present abnormal visual mental body image and overestimation of the size of tactile stimuli, with no differences between the magnitude of sensitivity and insensitivity of each body part. AN show a generalized inappropriate conceptualization of their own body [59]. Yet, one study which has implemented the one-point localization task involving the mapping of tactile stimulus onto a visual image, has found an opposite result. Women with AN and HCs did not differ in their performance in the way they show tactile processing and localization mechanisms [76].

In other studies which have implemented a Rubber Hand Illusion task (n = 2), AN patients show to have a stronger experience of ownership over the rubber hand in the task than healthy females. Thus, AN patients appear to have a more malleable and plastic body representation and more easily integrate a rubber hand into their body representation, most likely due to prioritizing external sensory input over interoceptive signals [61, 145]. Incidentally, in AN, hand location estimates are more influenced by external visual hand information and relatively less by proprioceptive hand information. Of note, body size and body shape, both aspects of body image, result also from the integration of visual and proprioceptive information [61, 145]. In another version of this kind of methodology, [62] applied a full body illusion task—so not on a specific body part like for the previous paradigm discussed—to prove possible improvements of body image disturbance through virtual reality visuo-tactile stimulation. The disturbed experience of body size in AN is flexible and can be changed, and this kind of method can denote a decrease of overestimation of highly emotional body parts.

AN perceive things differently based on body representations and beliefs concerning body size influence the specific somato-sensory process [119]. Data showed (n = 3) that the perceived bodies' dimensions affect the perception of the size of the tactile stimuli oriented in the altered body representation fashion. Importantly, the distortion in tactile processing found in AN patients is specific for spatial tactile perception and does not concern other dimensions of touch.

Nevertheless, in the opposite direction, it has been also pointed out how basic somato-sensory mechanisms of AN are not impaired, contrary to Keizer and colleague’s findings (2011). Taken together, these findings support the view of a selective impairment of AN on tasks tapping on a spatial representation of the body, and for a specific spatial dimension. Furthermore, AN patients perceive skin stroking as significantly less pleasant than HCs. No group differences could be found for the contrast between skin stroking and skin indentation in primary tactile regions. However, significantly less activity in the AN group in areas including left caudate nucleus as well as bilateral occipital cortex, showing both an affective and perceptual body image—body schema disturbances [19, 59]. Touch processing is not related to an aberrant elementary tactile mechanism, but to a more complex social, affective, and maladaptive mechanism related to brain reward and body image systems [19].

It was corroborated that anorexia patients present aberrant imagery processes not adopting motor strategies, resulting in an altered integration of multisensory bodily inputs (exteroceptive and interoceptive), based on the non-conceptual and pre-reflective representation of the bodily self. At an explicit level, comprising perceptual, psycho-affective, cognitive facets of own and other body image stimuli judgements, have been shown as altered only in AN patients [1]. Indeed, a mismatch between the actual size of participants’ hands and their stored (perceptual, cognitive, affective) body representation was found. It appears significantly easier for individuals with anorexia nervosa to respond to stimuli related to others rather than to oneself [1]. A high level of anxiety trait co-occurred during body performance and might therefore be an important clinical signature which outlines body image processing [46].

## Conclusions and future directions

In conclusion, from the multidimensional body image point of view, we can state that cognitive- emotional as well as somato-sensory components of body image should be considered to better understand embodiment experience in AN. Indeed, from the studies reviewed, it is clear how cognitive body representations/beliefs and emotional/interoceptive regulation strategies enter in a reciprocal relationship, influencing both perceptual and somato-sensory-somato-motor experience of own body perception [19, 34, 58, 101]. Thinking about body image distorted experience as a pure perceptual deficit is not sufficient to deeply understand this disorder-defining trait, and this awareness started to be evident from the very first less recent perceptual studies [34]. Thus, in future, behavioral and neuroimaging studies should focus on different limits as well as goals considering non-perceptual factors as important in shaping body image representation and body image-body schema interaction.

First, there are very few longitudinal studies which could shed a light on the influence of AN illness stage, age, and illness state-dependent features (e.g., BMI, nutrition) on the development of the embodiment experience. Second, it is important to engage a multi-modal approach design which can exploit the methodological benefits of different methods—neuroimaging (structural, functional, and electrophysiological ones), behavioral (considering all the dimensions) and physiological techniques. Indeed, when deepening the different limitations characterizing each component study, it is evident that the special and exclusive focus on each body image dimension prevents us from truly understanding it. More specifically, perceptual studies lack from a physiological counterpart deepening; the emotional studies are more unbalanced on these latter autonomic markers, with no conclusive or clear results; the cognitive research, instead, lack from a more specific physiological as well as neuronal perspective of body image; finally, for the somato-sensory ones, the same limitation of the cognitive studies can be drawn. Third, it is necessary to disentangle the physiological reactivity and cognitive-emotional roots interplay, due to physiological blunting issues which might shadow the real autonomic network system functioning as well as the influence of cognitive factors on emotional ones. Fourth, a more in-depth study of WR stage is needed, in order to make reliable and relevant comparisons with acute state body image distortion experience. Five, a more direct focus on the somato-sensory counterpart of the body image—body schema interplay could allow us to develop target-specific techniques on the structural and functional plasticity architecture which characterize AN patients. In conclusion, future studies should also specify better the subtype taken into account, as we have revised only a few corpus of studies which have clearly declared the subtype taken into account, with a predominance of the restrictive subtype. This is relevant in order to better outline subtle but significant neurobiological and psychological facets which allow to distinguish each anorexia's clinical profiles. We should acknowledge the need to realize a comprehensive study which can be used not only for diagnostic, but also for therapeutic purposes by putting in first line the cognitive, emotional, and somato-sensory counterpart of body image. This could offer patients the possibility of not an escape, but rather a different way of truly living their own body.

## Limitations

Several caveats should be outlined in this scoping review. First, this scoping review does not address a quantitative assessment of effect sizes as the studies here reviewed have implemented a wide range and heterogenous corpus of experiments. This limitation is valid also for accounting the statistical power of the original research. Second, the categorization of each study according to the involvement of each body image component (perceptual, cognitive, emotional and somatosensory/somatomotor ones) is not clear for every study, cause—as already stated in the method section—for some of these studies it was not possible to disentangle one component from the other in the primary involvement. In addition, there are not established guidelines for the categorization of body image dimensions studies. Third, we did not consider confounding factors such as the sample inhomogeneity (e.g., AN subtypes, state or duration of illness, age), the presence of psychiatric comorbidity and the pharmacological history in disentangle differences in how anorexia patients and healthy controls differently perceive their own and others body image stimuli. In conclusion, future literature reviews are needed in order to fill the gaps regarding body image as a multidimensional construct in anorexia nervosa.

## Supplementary Information

Additional file 1.

## Data Availability

No datasets were generated or analysed during the current study.
