# Supplementary material for: Behavioral, neuronal, and physiological facets of multidimensional body image in anorexia nervosa: a scoping review
Source: J Eat Disord. 2025 Feb 10;13:23. doi: 10.1186/s40337-025-01191-4 (PMC11812194; doi:10.1186/s40337-025-01191-4)
Supplement: Supplementary file 1 — Additional file 1. [file 40337_2025_1191_MOESM1_ESM.pdf]

Tables

Table 1: Body image behavioral studies reviewed

Paradigm	Authors	Sample	Diagnostic criteria	BMI<br>Age (SD)	Aims	Main findings
Visual size - estimation apparatus<br>*Cross-sectional and longitudinal study*	Slade and Russell, 1973	AN = 14 (13 females + 1 male, 12 inpatients)<br>HC = 20	Russell, 1970	n.r. [1]<br>19.79 (5.78)	Bodily perception: awareness of body dimensions	Overestimation of the width of one's body in AN. Pattern less marked when the judgments have to be for a female model. Weight restoration decreases the tendency: if persistence after weight gain, a subsequent relapse was more likely.
Distorting photograph technique + satiety aversion to sucrose test<br>*Cross-sectional study*	Garfinkel et al., 1978	AN = 26<br>HC = 16	Feighner et al., 1972	16.0 (n.r.)<br>20.08 (0.7)	Exploring body image awareness and interoception	Overestimation of body size and aversion to sucrose taste in AN. Neither viewing one's image in a mirror nor ingesting both "high" and "low" calorie connotation meals altered body size perception.
Visual size - estimation task and Image Marking task<br>*Cross-sectional study*	Pierloot & Houben, 1978	AN = 31 inpatients (13 schizoid + 7 hysterical)<br>HC = 20	Feighner et al., 1972	n.r.<br>20.92 (3.89)	Deepen body dimensions estimation process	Tendency to overestimate body size before treatment in AN.

1 N.r.: not reported data

		(neurotic manifestations)				
Visual size - estimation apparatus<br>*Cross-sectional study*	Casper et al., 1979	AN = 79<br>HC = 130	Russell, 1970	n.r.<br>n.r.	Investigate disturbance in body image and other disorder-traits relationship	Both AN and HC overestimated their body widths: overestimation cannot be considered unique to AN. Among AN patients, the degree of overestimation was associated with less weight gain during treatment, greater denial of illness, and several other pretreatment characteristics (poor outcome).
Distorting photograph technique, satiety aversion to sucrose test<br>*Cross-sectional study*	Garfinkel, et al., 1979	AN = 16<br>HC = 13	Feighner et al., 1972	n.r.<br>21.8 (0.86)	Exploring stability of perceptual disturbance after treatment	Anorexic subjects tend to overestimate body size and have an absence of aversion to repeated sucrose tastes. Stable disturbances.
Human figure drawing: Image Marking procedure<br>*Cross-sectional study*	Strober et al., 1979	AN = 18 inpatients (11 AN-r + 7 bulimic)<br>HC = 24 (neurotic, depressive and personality disorders)	Feighner et al., 1972	n.r.<br>14.77 (1.01)	Investigate body image functioning in first episode of AN	Size estimation is not a distinguishing variable, but experiences denoting estrangement from body, insensitivity to body sensations and weakness of body boundaries are more prevalent in AN. AN depicts a human figure with less differentiation relative to HC.
Video distortion, modified Image Marking Procedure and Body Image Screening Scale<br>*Cross-sectional study*	Meerman, 1983	AN = 36<br>HC = 35	Feighner et al., 1972	n.r.<br>18.86 (4.18)	Outlining perceptual body image disturbances	Concerning the video task, both groups show underestimation of their own body dimensions and of a female dummy. For the other techniques, an overestimation of body dimensions in AN patients (although not statistically different from other groups). AN shows significantly higher overestimation in

						both techniques for specific body dimensions (thighs, calves, and hips).
Lens (manipulate and create a horizontal distortion of an image projected into a monitor)<br>*Cross-sectional study*	Touyz et al., 1984	AN = 15 (middle phase)<br>HC = 15	DSM-III	n.r.<br>19.06 (5.18)	Exploring body shape perception	AN showed greater tendency to over and underestimate their present body shape. There is a trend in underestimating one's own body shape.
Video distortion, movable caliper and Image Marking tasks<br>*Cross-sectional study*	Fichter et al., 1986	AN = 21 (12 in acute and 9 chronic state)<br>HC = 12	Feighner et al., 1972	n.r.<br>20.2 (5)	Measure of body image disturbances	Significant overestimation compared to a control group in the variable's waist upper thigh, and in the compound measure soft body parts.
Video TV monitor distortion technique. Signal detection task<br>*Cross-sectional study*	Gardner & Moncrieff, 1988	AN = 9<br>HC = 9	DSM-III	n.r.<br>26.0 (n.r.);	Investigate whether body image is a sensory deficit	No differences in estimated actual body size between groups: for both groups, the ideal image was below the actual one. Underestimation pattern. No differences of sensory sensitivity between weight groups: AN more likely to report a distortion image of themselves, regardless the type of distortion used. Body image is no - sensory phenomenon.
Image Marking method<br>*Cross-sectional study*	Whitehouse et al., 1988	AN = 12 (8 AN-r, 5 inpatients + 7 outpatients)<br>HC = 20	DSM-III	n.r.<br>n.r.	Exploring body size estimation mechanism	Overestimation of waist size, but overall, no distortion of a body perception index for the whole body in AN.
Light beam apparatus<br>*Cross-sectional study*	Penner, Thompson &	AN-r = 13<br>HC = 26 (13	DSM-III-R	n.r.<br>n.r.	Body size overestimation	AN and HC did not differ in the extent to which they overestimated

	Coovert, 1991	with equal body sizes to AN)				their body size, but both displayed significantly greater overestimation than did the subjects of average size. Both groups also estimated the actual size of their body sites to be larger than average-size normal subjects
Video distortion method<br>*Cross-sectional study*	Probst et al., 1992	AN = 67<br>HC = 105	DSM-III-R	n.r.<br>21.99 (5.51)	Exploring body image assessment and ideal image	Both groups underestimate their body measures and only significantly differ on estimates of their ideal image: AN less underestimating their body image.
Nonverbal evaluation of body size (perception and motor dimensions): Askevold's "perception" test<br>*Cross-sectional study*	Molinari, 1995	AN = 20 inpatients<br>HC = 20	DSM-III-R	n.r.<br>n.r.	Evaluating the perceptive and motor dimensions of body image	AN overestimated the abdominal and pelvic area more relative to HC, during the projection of these are into space.
Female and male body drawings according to BMI<br>*Cross-sectional study*	Baluch et al., 1997	AN = 12 adults<br>HC = 16 teenagers<br>HC = 12 adults	DSM-III-R	n.r.<br>28 (1.64)	Exploring perception of body shape in teenage and mature AN patients	Anorexics rated fatter women less attractive, less healthy, less confident, and less popular. Rating male body shapes more attractive. Both anorexic and teenage groups rated the thinner women as more attractive than adult group.
Video distortion, Image marking and Kinesthetic Size tasks<br>*Cross-sectional study*	Lautenbacher et al., 1997	AN – WR = 23 (9 and 14 with intermediate and good outcome)<br>HC = 20 unrestrained eaters	DSM-III-TR	18.7 (1.4) for interme diate out.<br>20.8 (2.4)	Investigate perceptual body size estimation in restrained and unrestrained eaters	Using the Kinesthetic Size Estimation Apparatus, overestimation and uncertainty in the perception of body size became apparent in both groups of former patients. The other two methods, Video Distortion Technique and Image Marking Procedure, did not

		HC = 21 restrained eaters		for good out.<br>21.3 (2.1) for intermediate out.<br>23.9 (4.3) for good out.		produce comparable results.
Subjective Body Dimensions Apparatus-SBDA<br>*Cross-sectional study*	Gila et al., 1998	AN = 85<br>HC = 427	DSM-III-R	16.09 (0.02)<br>14.92 (1.70)	Deepen subjective body dimension perception	AN overestimated all parts of their body but their thorax, waist, and hips especially. For all parts of the body, the anorexic group showed greater overestimation.
Set of 64 anorexia related positive, negative and neutral words: encoding task, cued recall and word stem task<br>*Cross-sectional study*	Hermans et al., 1998	AN = 12 inpatients<br>HC = 12	DSM-III-R	15.04 (1.87)<br>21.08 (6.54)	Investigate implicit and explicit memory for ED related words	Strong explicit memory bias for anorexia related words; no differences in implicit memory.
Modified Stroop color-naming task with eating disorder relevant words + positive/negative valence words	Sackville et al., 1998	AN =20<br>HC = 33 with low dietary restrain<br>HC = 20 with high dietary	DSM-IV	15.02 (n.r.)<br>19.5 (2.9)	Exploring attentional biases towards eating disorder related words	Thinness and fatness words salient for AN, no preconscious attentional bias.

Cross-sectional study		restrain				
Computer based image distortion<br>*Cross-sectional study*	Hennighausen et al., 1999	AN = 36 inpatients<br>HC = 18	ICD-10	14.3 (1.4)<br>16.15 (1.95)	Is body image a perceptual deficit?	No general overestimation of body dimensions in AN in comparison to controls, but AN more often under or overestimated their body dimensions. Higher ideal body perception index and significant differences for specific body parts.
Body size estimation task<br>*Cross-sectional study*	Smeets et al., 1999	AN = 30 (in/outpatients)<br>HC = 28<br>HC thin = 36	DSM-III-R	17.74 (2.05)<br>23.80<br>(3.99)	Investigate body image disturbance: between sensitivity and biases	Signal detection analysis revealed no differences in perceptual sensitivity between groups; AN showed a bias to report thin differences. Body image disturbance as a reconstruction of visual body.
Morphing movie instrument<br>*Cross-sectional study*	Smeets, 1999	AN = 23 (11 AN + 12 EDNOS, 1 with past history)<br>HC = 12	DSM-IV	n.r.<br>n.r.	Body size<br>categorization	AN showed harsher judgments not only of their own body size and shape, but also of that of another woman model.
Proprioception test, Finger localization test, right-left orientation test<br>*Cross-sectional study*	Epstein et al., 2001	AN-r = 20 inpatients<br>HC = 20	DSM-IV	15.63 (2.43) pre-treatment<br>18.32 (1.26) post treatment	Neurocognitive processes behind body image disturbance: top-down or bottom-up?	Significantly differences only during pre-treatment regarding executive functions and body-schema functions. Subtle cognitive dysfunction not specific but which can interact with body schema. Body schema as a top-down process in AN.

				n.r.		
Divided visual field experiment<br>*Cross-sectional study*	Smeets & Kosslyn, 2001	AN = 22 (10 AN + 12 EDNOS, 12 past history of ED + 1 acute)<br>HC = 23	DSM-IV	16.7 (1.1) in present AN<br>20.3 (2.1) in past AN<br>27.8 (n.r.)	Assessment of hemispheric differences of body image	AN participants judged a higher proportion of fatter distortions as equal to their own size. They responded faster when stimuli were presented initially to the LH than when they were presented initially to the RH. In contrast, fewer thinner distortions were judged as equal to their own body size and were judged more slowly on LH trials than on RH trials. No hemispheric differences when judging pictures of somebody else.
Stroop Task Revised<br>*Cross-sectional study*	Fassino et al., 2002	AN-r = 20<br>HC = 20	DSM-IV	15.6 (2.2)<br>23.8 (6.6)	Attentional biases and frontal functioning	Disturbances of body image might be linked to the alterations of abstraction and critical abilities as well as with an obsessive frontal functioning in AN.
Computer based Body Image Distortion tool (Q-BID)<br>*Cross-sectional study*	Roy & Forest, 2007	AN-r = 22 outpatients<br>AN-r = 18 inpatients<br>HC = 200	DSM-IV	n.r.<br>15.64 (1.51) outp.<br>15.37 (1.52) inp.	Assessment of BID	Significant body image distortion differences between anorexic and non-anorexic teenager girls. Differences between inpatient and outpatient body image distortions: overestimation in acute state more relevant relative to the weight restored one.
Video distortion technique<br>*Cross-sectional study*	Urdapilleta et al., 2007	AN = 22 inpatients<br>HC = 22<br>Jazz dancers = 22	DSM-IV	15.7 (1.3)<br>21 (2.4)	Investigate body image distortions	All three groups perceived themselves to be heavier than they actually are: the dancers had a more realistic perception of their body weight, would have liked to be thinner, whereas the controls and anorexics were satisfied with their

						body weight; the scores of the anorexic subjects lacked correlation between perceptual and idealized distortions, relative to others groups.
Affective Priming Task<br>*Cross-sectional study*	Cserjési et al., 2010	AN-r = 35<br>HC = 35	DSM-IV-TR	14.76 (1.31)<br>19.61 (3.42)	Body shape evaluation on both automatic and controlled level	In contrast to the control group, AN did not show a positive attitude toward the ultra-thin body shape on the automatic level. The AN evaluated the overweight body as negative group both on the automatic and the self-reported levels.
Anticipation of body scaled action<br>*Cross-sectional study*	Guardia et al., 2010	AN = 25<br>HC = 25	DSM-IV	15.14 (1.55)<br>24.32 (6.54)	Nature of body image biases	The anticipation of body-scaled action is severely disturbed in AN. The abnormally high 'passability ratio' (the critical aperture size to shoulder width ratio) correlated with the duration of illness and the degree of body concern/dissatisfaction. Neural processing in parietal networks should be considered.
Psychophysical procedure based on perception (approaching visual stimulus)<br>*Cross-sectional study*	Nico et al., 2010	AN = 8<br>HC = 11<br>Right-handed with vascular lesion = 4	DSM-IV	15.3 (2.1)<br>23.4 (4.0)	The role of parietal lobe on body image disturbance	Healthy volunteers and left parietal patients estimated body boundaries very close to the real ones. Conversely, anorexics and right parietal patients underestimated eccentricity of their left body boundary. Role of parietal cortex in AN pathogenesis.
Morphing technique: body size and attractiveness rating with eye-tracking<br>*Cross-sectional study*	George et al., 2011	AN = 16<br>HC = 16	DSM-IV	16.8 (2.1)<br>26.2 (7.9)	Attentional biases influence on eye movement and visual information	AN observers overestimate body size relative to controls and find bodies with lower body mass indexes more attractive. Wider fixation patterns encompass the prominence of the hip, collar bones, and stomach.

					processing	Alteration of visual processing and overestimation of body size.
Tactile Estimation Task (TET) and Distance Comparison Task (DCT)<br>*Cross-sectional study*	Keizer et al., 2011	AN = 20 (5 EDNOS)<br>HC = 25	EDE	18.54 (2.03)<br>22.30 (3.01)	Deepen somatosensory aspects of body image	Less accuracy in visualizing own bodies and overestimation of distances between tactile stimuli (disturbance in visual and sensory body image) in AN.
Door like varying in width aperture task<br>*Cross-sectional study*	Guardia et al., 2012	AN = 25 (12 AN-r + 13 AN-b/p)<br>HC = 25	DSM-IV	15.64 (1.2)<br>23.84 (7.75)	Examine abnormal representation of the body in action	Higher passability ratio in AN patient for first perspective. Impairments of the overall network involved in the emergence of the body schema and in one's own perspective judgments.
Delayed matching to sample tasks (body action and form discrimination task)<br>*Cross-sectional study*	Urgesi et al., 2012	AN = 12 (7 AN-r, 2 AN-b/p, 2 EDNOS)<br>HC = 15	DSM-IV-TR	17 (1.8)<br>20.8 (7.7)	Deepen visual body perception deficits of others	Better visual discrimination performance in detail-based processing of body forms in AN, but not of body actions. The paradoxical advantage of patients with AN in detail-based body processing associated with the tendency to routinely explore body parts (obsessive worries about body appearance).
Standardized BMI pictures of own/young women models: eye movements analysis	von Wietersheim et al., 2012	AN = 35<br>HC = 32	EDI-2	16.4 (n.r.)<br>22.9 (n.r.)	Investigate attention and biases for body images	Patients with AN judge their own body areas as being less attractive than the controls. AN were also more critical in their assessment of the bodies of others. They spent less

and attractiveness rating scale<br>*Cross-sectional study*						time looking at their own breasts, but more time at their thighs. Cognitive biases as predictive factors.
Aperture Task<br>*Cross-sectional study*	Keizer et al., 2013	AN = 19 (13 AN + 6 EDNOS)<br>HC = 20	EDE	18.32 (2.69)<br>23.68 (4.62)	Unconscious perceptual processes behind body schema	Abnormalities in AN at the level of the unconscious, action-oriented body schema. Body representation disturbances in AN are pervasive: they do not only affect (conscious) cognition and perception, but (unconscious) actions as well.
Rubber Hand Illusion (RHI)<br>*Cross-sectional study*	Keizer et al., 2014	AN = 30 (10 EDNOS)<br>HC = 30	EDI-2	17.50 (2.14)<br>26.37 (9.08)	Exploring body image disturbances in size perception	Stronger experience of ownership over the rubber hand in the AN group: more malleable body representation. Changed hand size estimation in the AN group appears to be unrelated to the RHI.
Body size morphing program<br>*Cross-sectional study*	Øverås et al., 2014	AN = 37 (20 EDNOS)<br>HC = 35	DSM-IV	n.r.<br>18.8 (3.96)	Investigate body size disturbances in memory versus perception	Overestimation of body size both in memory and perception condition for AN. Anxiety plays a role in body size disturbances.
Visual scanning behaviour task: body image shape competition with social interaction themes. Eye-tracking method<br>*Cross-sectional study*	Pinhas et al., 2014	AN = 13 (10 AN-r + 3 AN-b/p)<br>HC = 20	DSM-IV	n.r.<br>14.5 (1.61)	Investigate attentional biases to body shapes	When images of thin body shapes are presented along with social interactions, more visual scanning at TBs rather than social interactions, same for fat body shapes in AN. Hierarchy of attentional allocation to TBS images, followed by fat and social interactions ones during all stimuli presentation.

Affective standard pictures and body emaciated pictures. Startle response reflex.<br>*Cross-sectional study*	Reichel et al., 2014	AN = 36 (inpatients + outpatients)<br>HC = 36	ICD-10	15.8 (1.4)<br>15.9 (2.0)	Investigate appetitive reaction to body emaciated pictures	Significant group difference with a startle inhibition (appetitive response) among patients, and a startle potentiation (aversive response) among the controls, whereas no such difference for subjective measures was found.
Affect misattribution procedure (pleasant, neutral, unpleasant food – weight relevant images)<br>*Cross-sectional study*	Spring & Bulik, 2014	AN = 9<br>AN-WR = 14<br>HC = 29	DSM-IV	n.r.<br>21.4 (5.79)	Deepen implicit and explicit affect toward food and weight stimuli	Greater implicit negative affect toward unpleasant overweight body type images in acute AN state.
Photographs of other's bodies.<br>Emaciation condition and control condition. Lexical decision task (LDT)<br>– word recognition task<br>*Cross-sectional study*	Smith et al., 2014	AN = 30<br>HC = 29	DSM-5	17.51 (1.03)<br>20.03 (2.83)	Exploring implicit attitudes towards emaciation and thinness	At an implicit, automatic level, AN had stronger association between emaciation and both beauty and ugliness than control, suggesting that women with AN may have atypical beliefs about beauty. Thin ideal internalization is an important factor in the development and maintenance of eating disorders.
Inversion effect paradigm<br>*Cross-sectional study*	Urgesi et al., 2014	AN = 12 (7 AN-r + 2 AN-b/p + 3 EDNOS)<br>HC = 12	DSM-IV-TR	17 (1.8)<br>20.8 (7.7)	Investigate impairment in the configural body processing	Selective deficits in the discrimination of upright body stimuli (configural processing), but not in detailed-based processing images in AN. Obsessive worries about body appearance and excessive attention to details characterize general perceptual style.
Body size distortion	Hagman et	AN = 74	DSM-IV	15.98	Investigate	Overestimation of body size in AN is

and dissatisfaction apparatus<br>*Cross-sectional study*	al., 2015	HC = 11		(1.20)<br>15.95 (2.29)	body size overestimation and attitudes	related to the psychopathology associated with the disorder, and it is not due to any perceptual tendency for people with lower BMI to overestimate their body size.
Body image morphing technique<br>*Cross-sectional study*	Horndasch et al., 2015	AN = 20 adolescents<br>AN = 19 adults<br>HC = 37 adolescence group	ICD-10<br>DSM-IV	16.1 (1.6) in adolescents<br>15.6 (1.8) in adolescents<br>16.5 (2.0) in adults<br>25.3 (5.1) in adults	Investigate perception and evaluation of different BMI body images	AN patients estimated other women's weight higher. Bias towards assessing extremely underweight women as more attractive and normal weight and overweight women as less attractive. Effects are more pronounced in the adult group. Attractiveness bias towards strongly underweight others' bodies, and the interaction of this bias with age, as predictors.
Von Frey's test and Two-point discrimination threshold, distance task and time duration task<br>Body image test - Daurat-Hmeljak task<br>*Cross-sectional study*	Spitoni et al., 2015	AN = 18<br>HC = 32	DSM-IV-TR	16 (1.3)<br>24.8 (3.71)	Investigate somatosensory components of body image perception	Patients judged horizontal tactile stimuli significantly wider than the same stimuli oriented vertically. These results suggest that AN perceive things differently based on body representations and that the beliefs concerning body size influence the specific somatosensory process of tactile experience.
Exposure to thin or round body pictures<br>*Cross-sectional study*	Cazzato et al., 2016	AN =13 (4 AN-r + 9 AN-b/p)<br>HC = 13	DSM-IV-TR	16.44 (0.52)<br>26.92	Effects of body exposure on self-body image and aesthetic	AN patients' perception of their own and others' body is more easily malleable by exposure to round figures as compared to controls.

				(1.70)	appreciation	Crucially, this mechanism may strongly contribute to the development and maintenance of self-body image disturbances.
Body size estimation task<br>*Cross-sectional study*	Cornelissen et al., 2016	AN = 20 (10 inpatients + 10 outpatients)<br>HC = 20 with normal level of body shape concern<br>HC = 20 without a normal level of body shape concerns	DSM-5	21.71 (3.95)<br>23.70 (4.43)	Exploring patterns of eye movements during body size estimation	Accurate body size estimators tended to look more in the waist region, independent of clinical diagnosis; there is a pattern of looking at images of bodies, particularly viewing the upper parts of the torso and face, which is specific to participants with AN but which is independent of accuracy in body size estimation. Distributed fixation patterns.
Full body illusion task (FBI): estimation of body size before and after VR synchronous visuo-tactile stimulation<br>*Cross-sectional study*	Keizer et al., 2016	AN = 30 (6 EDNOS)<br>HC = 29	DSM-IV	18.11 (1.68)<br>22.03 (3.67)	Improvements of body image disturbance through VR	The disturbed experience of body size in AN is flexible and can be changed: decrease of overestimation of highly emotional body parts.
Exposition to thin or round bodies models<br>*Cross-sectional study*	Mele et al., 2016	AN = 20 (16 AN-r + 4 AN-b/p)<br>HC = 20	DSM-IV-TR	16.57<br>(2.06)<br>15.45 (1.75)	Investigate altered exposure-related shaping of body appreciation	Brief exposure to round models increased liking judgments of thin body models in AN. Weak norm- based reshaping of body appreciation.
Biological motion	Phillipou et	AN = 24	DSM-5	16.52	Investigate	Hyper scanning behavior regarding

task and eye tracking<br>*Cross-sectional study*	al., 2016	HC = 24		(1.14)<br>23.07 (6.88)	accuracy in perceive the size of other's bodies	stimuli in AN, but not differences from how visual attention is directed to different body areas relative to HC. No differences in body size estimations between groups. Body image disorders are related only to one's own body.
Mood induction and mirror exposure methods. Eye-tracking.<br>*Cross-sectional study*	Svaldi et al., 2016	AN = 12 (5 AN-r, 5 AN-b/p, 2 EDNOS)<br>HC = 12	DSM-IV	18.13 (1.46)<br>15.14 (1.55)	Relationship between mood and selective attention to body image	After negative mood induction, increased selective attention to most ugly vs beautiful body parts, with stronger gaze frequency in AN. Negative mood contributes to the maintenance of pathological information processing of the self-body.
Rubber Hand Illusion (RHI)<br>*Cross-sectional study*	Zopf et al., 2016	AN = 23<br>HC = 23	DSM-5	15.82 (1.27)<br>21.87 (2.79)	Investigate processing of multisensory bodily signals	Significant group differences in shift of reaching trajectories: proprioceptive signals on hand location are reduced in AN (biases towards external visual information). Multisensory body location perception changes in AN.
Biometric Figure Rating Scale (FSR)<br>*Cross-sectional study*	Mölbert et al., 2017	AN = 24 (19 AN-r + 5 AN-b/p)<br>HC = 104	ICD-10	15.07 (1.62)<br>23.29 (5.66)	Novel perceptual paradigm for body image	In FRS, AN patients desired a body close to their actual BMI. No visual disturbances behind body image deficits.
Photographs of others faces and bodies (rating tasks)<br>*Cross-sectional study*	Moody et al., 2017	AN = 22 (inpatients, 20 females and 2 males)<br>BDD = 30	DSM-IV	20.3 (1.3)<br>21.5 (4.5)	Appearance evaluation of others bodies	AN showed lower attractiveness, body size overestimation pattern and trigger for their own bodies. More complex cross-disorder body image phenotype.

		HC = 39				
Scrambled Sentences Task<br>*Cross-sectional study*	Brockmeyer et al., 2018	AN = 40<br>HC = 40	DSM-5	16.80 (1.74)<br>23.73 (1.94)	Examine negative interpretations biases towards own body	AN showed a stronger body-related negative interpretation bias than HC. Within both groups, negative interpretation bias correlated strongly and positively with AN symptom severity and these effects were not moderated by levels of depressive symptoms. Biased interpretation of body-related information is associated with the specific psychopathology of AN.
One-point-localization (OPL) task<br>*Cross-sectional study*	Mergen et al., 2018	AN = 27 (2 atypical)<br>HC = 40	DSM-5	15.70 (1.04)<br>27.44 (9.20)	Evaluating visuo-tactile body perception	Women with AN and HC did not differ in their performance. Both groups showed systematic distortions in their localization performance. Mapping of a tactile stimulus does not involve a distorted body representation in women with AN compared to HC.
Three-dimensional body scan<br>*Cross-sectional study*	Mölbert et al., 2018	AN = 24 (23 inpatients + 1 outpatient)<br>HC = 24	DSM-5	15.17 (1.47)<br>24.00 (6.35)	Body image disturbance: perceptual and attitudinal components	Contradiction to the assumption that patients with AN overestimate their body weight due to visual distortions. Rather, BID might be driven by distorted attitudes regarding the desired body.
Adjectives primes and body images (size-estimation task)<br>*Cross-sectional study*	Kazén et al., 2019	AN = 20 (8 AN-b/p)<br>HC = 22	ICD-10	18.9 (1.81)<br>26.5 (9.88)	Body image perceptions: right hemispheric activation treatment	The distorted "fatter than" body image was found only in AN and only when a picture of their own body appeared on the RVF and was preceded by negative self-relevant words. This distorted perception of the patients' body image was reduced after left-hand muscle contractions

						(right hemispheric activation).
Virtual reality and multisensory body illusion (interpersonal multisensory stimulation)<br>*Cross-sectional study*	Provenzano et al., 2019	AN-r = 20<br>HC = 20	DSM-5	15.86 (1.12)<br>23.30 (7.61)	Characterize and reduce the perceptual, the cognitive-emotional components BID	Higher body dissatisfaction in AN, but not body size overestimation. The cognitive-emotional, more than the perceptual component of BID, is severely altered in AN and that perspective from which a body is evaluated may play a crucial role. No reduction of BID after the interpersonal stimulation.
Approach Avoidance Task<br>*Cross-sectional study*	Brockmeyer et al., 2020	AN = 39 (22 AN-r, 17 AN-b/p)<br>HC = 38	DSM-5	16.79 (1.76)<br>23.87 (6.97)	Investigate approach and avoidance bias for body shapes	No differences in the automatic approach–avoidance tendencies towards thin-ideal and normal-weight bodies. Less positive implicit evaluation of thin other women in AN, and an implicit preference for thin bodies depicted as themselves.
Picture of own's bodies + standardized obese one: eye-tracking study<br>*Cross-sectional study*	Hartmann et al., 2020	AN = 26<br>HC = 16	DSM-5	15.76 (2.70)<br>23.28 (7.59)	Cognitive biases related to attentional system	For both stimuli, participants focused longer on the subjectively unattractive body parts, even stronger attentional bias in individuals with AN (obese stimulus). Both groups also gaze longer at body parts indicative of weight status or gain.<br>The attentional bias to one's own subjectively unattractive body parts might represent a mechanism maintaining body image disturbance in women in general. Attentional biases are even stronger in AN.
Full-body illusion (FBI) with visuo-motor and visuo-tactile stimulation	Porras et al., 2020	AN = 30<br>HC = 43 (25 with low body dissatisfaction	DSM-5	17.55 (1.07)<br>17.73	Usefulness of VR in assessing cognitive and emotional body	AN patients reported higher fear of gaining weight, body anxiety and body-related attentional bias. AN reported significantly lower FBI

(VR)<br>*Longitudinal study*		and 18 with high BD)		(4.60)	image processes	levels relative to HC.
Door like aperture varying in width task<br>*Cross-sectional study*	Beckmann et al., 2021	AN = 21 (5 AN-b/p + 18 AN-r)<br>HC = 21	EDE-Q	14.60 (1.88)<br>24.67 (5.61)	Investigate distortions in body schema	Significantly higher critical aperture to shoulder ratio for AN, unconsciously estimating their body size to be larger than in reality. Correlation between negative body attribution and overestimation of bodily dimensions.
Weight bias task: adjectives - BMI bodies matching task<br>*Cross-sectional study*	Behrens et al., 2021	AN = 39 (20 atypical)<br>HC = 40	ICD-10	AN 16.17 (1.21)<br>atypical<br>18.87<br>(1.33)<br>AN<br>23.82<br>(8.31)<br>atypical<br>26.55<br>(7.04)	Exploring weight bias and linguistic body representation	Patients with both typical and atypical AN affectively and visually represent body descriptions not differently from HC. Overvaluation of low body weight and fear of weight gain cannot be explained by generally distorted perception or cognition but require individual consideration.
Implicit association test: picture based implicit association test, questionnaire based IAT<br>*Cross-sectional study*	Borgers et al., 2021	AN = 35<br>HC = 29	DSM-IV	15.87<br>(2.85)<br>24.80<br>(9.23)	Deepen fear of gaining weight and implicit drive for thinness evaluation	Correlation analyses within the AN group showed that higher implicit drive for thinness was associated with more pronounced eating disorder-specific psychopathology, in both IATs. Greater implicit drive for thinness and fear of gaining weight as continuous construct.
Perceptual size estimation task	Brown et al., 2021	AN = 19	DSM-IV-TR	16.90 (1.05)	Body size overestimation:	Significantly overestimate body size across blocks in AN. Significant

basing on different sensory information<br>*Cross-sectional study*		HC = 19		16.9 (2.24)	cognitive, affective, tactile, and visual information	correlations between body size overestimation and drive for thinness and body dissatisfaction in the AN. Results suggest that both deficits in tactile, visual perception and affective factors play a role in BID for young women with AN.
Mirror Gazing Test (MGT)<br>*Cross-sectional study*	Demartini et al., 2021	AN = 14 (outpatients)<br>HC = 14	DSM-5	15.5 (1.3)<br>28.3 (10.7)	Visual perception-dissociations processes behind body image	Dissociative sensations during MGT: dissociative identity and depersonalization were much higher in patients with AN. These findings were correlated with body dissatisfaction and disruption in interoceptive awareness.
Visual Estimation Task, Tactile Estimation Task and Hoop Task<br>*Cross-sectional study*	Hasenack et al., 2021	AN = 29<br>HC = 30	DSM-5	16.79 (3.03)<br>22.00, (3.37)	Investigate the link between anxiety and body attitudes - body size estimation	State anxiety levels increased significantly more in AN patient than in HC after completing each task.<br>Thus, performance of AN patients on BSE and other body-related tasks might not just indicate the (mis)perception of their body but also co-occur with increased state anxiety.

Skin conductance measurement (SCR) during rest, neutral and mirror stimulus exposure<br>*Cross-sectional study*	Knejzlíková et al., 2021	AN-r = 30 (4 atypical type)<br>HC = 30	DSM-5	16.9 (3.73)<br>14.9 (1.31)	Investigate EDR during mirror exposure	AN rated mirror exposure as a subjectively more stressful experience. Differences in skin conductance response (SCR) were not significant; variance in SCR was substantially greater in AN. The overall (SCL) was lower in AN. Increase in SCR during mirror exposure, as opposed to exposure to neutral stimuli, was positively related to the tendency to experience negative emotions, interoceptive sensitivity, body dissatisfaction and suppression.
Digital tool, "Somatomap" to explore BID<br>*Cross-sectional study*	Ralph-Nearman et al., 2021	AN = 55<br>HC = 55	DSM-5	18.85 (2.83)<br>25.25 (11.00)	Exploring visual mapping of body image	The AN group demonstrated greater regional perceptual inaccuracy for their current body, greater discrepancies between their current and ideal body, and higher body dissatisfaction. Body concerns are localized disproportionately to the chest and lower abdomen.
Pupil psychosensory reflex (PSR) in response to standardize – own body silhouettes<br>*Longitudinal study*	Couton et al., 2022	AN = 42 (23 inpatients, 13 AN-b/p, 29 AN-r)<br>HC = 35	DSM-5	15.66 (1.35)<br>26.76 (7.56)	Exploring PSR to different body silhouettes	PSR correlated with the subjective rating of emotional arousal in controls, not in patients. With pictures of their own silhouettes, pupil response to underweight stimuli differed from pupil response to normal weight or overweight stimuli in both groups. Weight gain was associated with an increase in PSR, an improvement of ideal BMI and a decrease of body dissatisfaction: no change in perceptual distortion.

Body image distortion method with different proxies of physical exercise and rating scale for own body image after standardized effort test<br>*Cross-sectional study*	Di Lodovico et al., 2022	AN = 40<br>HC = 21	DSM-5	16.78 (2.31)<br>23.42 (5.59)	Investigate relationship between body image disturbance and physical exercise	Physical exercise may contribute to the distortion of body image in AN and explain the paradoxical augmentation of unhealthy exercise despite ongoing weight loss.
Viewing pictures depicting negative, neutral, standardized and non-ED positive stimuli; AMSR paradigm ED stimuli<br>*Cross-sectional study*	Mahr et al., 2022	AN-WR= 7 (AN-r)<br>AN = 14 (acute state, AN-r)<br>HC = 7	DSM-5	15.19 (1.74) in AN<br>19.27<br>(1.68) in AN-WR<br>13.46 (1.66) in AN<br>16.00 (0.89) in AN-WR	Investigate affective modulation of startle response during ED stimuli	All groups showed an inhibition of startle response to the four ED-related categories. In contrast, AN and AN-WR showed reduced hedonic response to standardize positive stimuli (correlation with social anxiety related traits). Early automatic responses to ED stimuli do not differ from HC.
Accuracy and TRs during two validated task (explicit and implicit recognition of self/other hand stimuli)<br>*Cross-sectional study*	Ambrosecchia et al., 2023	AN-r = 25 (inpatients)<br>HC = 27	DSM-IV	16.1 (0.3)<br>23 (1.9)	Exploring bodily self-recognition and overestimation with their implicit/explicit mechanisms	The perceived size of hand stimuli modulated both the implicit and explicit processing of body parts in both groups; the implicit self-advantage emerged in both groups, but the bodily self, at an explicit level (perceptual, psycho-affective, cognitive) together with the integration and the distinction between self and other, was altered only in AN.

Flanker test revised<br>*Cross-sectional study*	Fusco et al., 2023	AN = 25<br>HC = 25	DSM-5	16.64 (0.21)<br>21.60 (1.20)	Processing of conflicting body representations and cognitive control	A dysfunctional association between the processing of body-related representations and cognitive control mechanisms in AN.
Body size estimation paradigm<br>*Cross-sectional study*	Gadsby et al., 2023	AN-r = 14<br>HC = 40	DSM-5	16.2 (1.84)<br>23.8 (4.89)	Testing visual misperception hypothesis on body image	Both women with AN and women without any eating disorder overestimate their body size. In the transposed task, neither group adjusted the bodies to be narrower than the rectangle. Participants with AN set their photographs to be significantly wider. The results don't confirm overestimation stems exclusively from visual misperception: substantial response bias effect.
Viewing of pictures with different weight categories and HR registration<br>*Cross-sectional study*	Horndasch et al., 2023	AN = 37 (19 adolescents and 18 adults, inpatients)<br>HC = 43 (18 adolescents and 25 adults)	DSM-5	15.9 (1.5) for adolescents<br>16.5 (1.8) for adults<br>15.5 (1.9) for adolescents<br>27.3 (7.9) for	Exploring physiological emotional markers of body perception	Differential HR reactions for anorexia nervosa patients and HC were found for body stimuli in general. The highest HR decelerations in response to pictures of strongly underweight and overweight women may reflect emotional processes - such as anxiety due to social comparison - in AN.

				adults		
B-BART (Balloon Analogue Risk Task) with body avatars<br>*Cross-sectional study*	Jenkinson et al., 2023	AN-r=31<br>AN-WR=2<br>3 HC=485	DSM-5	16.5-18.5 (n.r)<br>n.r	Transdiagnostic and computational approach to explore eating restriction decisions	Social and motivational values regarding body appearance influence value-based, decision making in eating restriction. These behaviors are driven by an aversion to risk rather than loss, with desirable body outcomes being associated with less risk aversion, and undesirable body outcomes linked to greater risk aversion.
Go-No-Go Association task<br>*Cross-sectional study*	Lakritz et al., 2023	AN = 28 (binge/purging subtype; restrictive subtype)<br>HC = 29	EDI-II	16.70 (1.5)<br>23.10 (4.7) for first recruit ment<br>16.10 (1.4)<br>27.60 (6.6), for second recruit ment	Implicit associations between food and bodily stimuli	The AN group tended to categorize stimuli as low-calorie foods and underweight bodies less than the HC group, and they tended to categorize stimuli as high calorie foods and overweight bodies more than the HC group. Implicit associations support body image disturbance in AN.
Linguistic embodied task (association between body action picture and written verb)<br>*Cross-sectional study*	Meneguzzo et al., 2023	AN = 45<br>Atypical = 43<br>HC = 55 + 24 subsample	DSM-5	14.72 (1.82) in AN<br>20.55<br>(1.18) in atypical<br>22.29	Exploring linguistic embodiment in AN	Both the clinical groups showed an abnormal eating disorder ability to evaluate the image-word matching and needed longer TRs. Impaired embodied cognition in AN.

				(5.79) in AN<br>22.67<br>(5.19) in atypical		
Explicit motor imagery, mental rotation test and visuospatial perspective task<br>*Cross-sectional study*	Meregalli et al., 2023	AN = 52 (inpatients)<br>HC = 62	DSM-5	15.87 (1.31)<br>19.10 (4.21)	Investigate body schema and motor imagery as proxies for body image deficits	Greater difficulties in imagining movements according to a first-person perspective, lower accuracy in motor imagery, selective impairment in the mental rotation of human figures, and reduced ability in assuming a different egocentric visuospatial perspective in AN.
Anxiety induction task (pictorial dot-probe task with different BMi body-related pictures or non-disorder-relate d threatening pictures)<br>*Cross-sectional study*	Radix et al., 2023	AN = 23<br>HC = 29<br>MDD = 27	DSM-5	16.52 (1.62) in AN<br>24.23 (5.18) in MDD<br>15.06 (1.29) in AN<br>15.78 (1.12) in MDD	Interaction between anxiety-attentional biases with eating disorder traits	The anxiety induction did not affect the observed attention pattern. AN showed an AB towards underweight body pictures compared to HC, whereas no disorder-unspecific threat-related AB emerged. Only anxiety predicted the AB towards underweight body pictures.
Interactive 3D body image morphing tool	Schloesser et al., 2023	AN-WR = 33 (24 women and 9 males)	DSM-5	21.3 (2.7) in AN	Sex-specific characteristics of long-term	Male remitted patients demonstrated significantly stronger muscularity-focused body image ideals, evident

Longitudinal study		HC = 36		female2 2.9 (3.5) in AN males<br>22.0 (3.2) in female 24.1 (4.4) in males	remitted AN with respect to residual eating disorder (ED) psychopathology, body image, and endocrinology.	in interviews, self-reports, and behavioral data, than both female patients and HCs.

Table 2: body image neuroimaging studies reviewed

Paradigm	Authors	Sample	Diagnostic criteria	BMI<br>Age (SD)	Aims	Main findings
Computer based life image distortion technique: fMRI task-based design<br>*Cross-sectional study*	Seeger et al., 2002	AN = 3<br>HC = 3	DSM-IV	15.3 (0.6)<br>17.0 (0.5)	Exploring the body image effects on amygdala	Higher activation of right amygdala, right fusiform gyrus and brainstem regions when AN are exposed to their own body image: "fear network" involvement in body image biases.
Body distortion-morphing technique:	Wagner et	AN = 13 (10 AN-r +	DSM-IV	14.6	Investigate neural	Greater activation in the prefrontal cortex and the inferior parietal lobule,

fMRI task-based design<br>*Cross-sectional study*	al., 2003	3 AN-b/p)<br>HC = 10		(1.3)<br>15.3 (1.4)	activity changes during body distortion process	including the anterior intraparietal sulcus in AN. Specific increase in activation to their own pictures than to others indicating different visuo-spatial processing in AN.
Perception of own and other's body images: fMRI task-based design<br>*Cross-sectional study*	Sachdev et al., 2008	AN = 10 (5 AN-r + 5 AN-b/p)<br>HC = 10	DSM-IV	16.40 (1.03)<br>18.8 (1.69)	Processing of own's body images vs other's one's neural correlates	Processing of no self-bodies' images imply the activation of inferior and middle frontal gyri, superior and inferior parietal lobules, posterior lobe of the cerebellum and thalamus in a similar pattern relative to HC, but with greater activation of medial frontal gyrus in clinical sample. No greater activation of any region in differential self-no self-comparisons in AN. Self-images are processed in a quite differential way (lack of activation of attentional system and insula), but the non-self-showed similar pattern relative to HC.
Visual stimuli of bodies images according to different BMI: an fMRI task-based design<br>*Cross-sectional study*	Fladung et al., 2010	AN = 13 (9 AN-r + 5 AN-b/p)<br>HC = 14	DSM-IV	16.6 (1.2)<br>16.0 (1.8)	Ventral striatum reward system and body image	Women with AN provided significantly higher positive ratings in response to underweight stimuli than in response to normal-weight stimuli, Ventral striatal activity demonstrated a highly significant group-by-stimulus interaction for underweight and normal-weight stimuli. AN neuronal activations were higher during processing of underweight stimuli compared with normal-weight stimuli.
Body shape images from slim models and interior design images: fMRI task-	Friederich et al., 2010	AN = 17<br>HC = 18	DSM-IV	15.6 (1.4)<br>24.9	Neural correlates of body dissatisfactio	Greater anxiety to the self-other body-shape comparison in AN, as well as less satisfaction with their current body shape. The self-other body-shape

based design<br>*Cross-sectional study*				(5.6)	n	comparison induced more activation of the right sensorimotor brain regions (insula, premotor cortex) and less activation of the rostral anterior cingulate cortex (ACC). Critical role of insula hyperactivation and anterior cingulate cortex hypoactivation for altered thin models drivenness in AN.
Satisfaction rating and size estimation of own body size – distorted photographs: fMRI task-based design<br>*Cross-sectional study*	Mohr et al., 2010	AN = 16<br>HC = 16	DSM-IV	15.9 (1.25)<br>24.1 (3.4)	Separating neural component of body image	AN showed less satisfaction with their current body size (PrC/PPC) and stronger activation of IS, PFC rating thin self-images (emotional involvement).
Valence Rating task, working memory task: fMRI task-based approach<br>*Cross-sectional study*	Pruis et al., 2012	AN-WR= 15<br>HC = 16	DSM-IV	21.5 (n.r.)<br>39.0 (n.r.)	Frontal lobe emotional regulation and body image	Negatively rated images were more disruptive to working memory than neutral or positively rated images in both groups; however, amygdala and fusiform activation were greater in women who had recovered from AN than in controls when viewing images of bodies during the working memory task. There were no group differences in lateral prefrontal activity. Yet, there was more suppression of medial prefrontal cortex activity in AN-WR in comparison to controls when negatively rated images were presented during the working memory task. Development of neural compensating processes that prevent emotional responses from disturbing cognition.

View of own body images (normal size and distorted): fMRI task-based design<br>*Cross-sectional study*	Castellini et al., 2013	AN-r = 18<br>HC = 19	DSM-IV	16.07 (1.42)<br>24.74 (7.58)	Conflicting results for neural correlates behind BID	Widest extent of activation in the oversize condition in AN relative to controls. Similar pattern of activation to the view of one's body, with an increase in the EBA, superior and inferior parietal lobule and PFC. More pattern of activation in DLPFC in response to oversized body picture (correlation to ED symptoms) in AN.
Grey scale photographs of bodies and neutral stimuli: fMRI task-based design<br>*Cross-sectional study*	Suchan et al., 2013	AN = 10<br>HC = 15	DSM-IV	15.7(1.5)<br>26 (9.0)	Connectivity network between EBA and left fusiform area	Left sided effective connectivity in the occipital cortex of women with AN showed a highly negative correlation with body size misjudgments. Present results yield evidence for altered networks for body processing in women with AN. Results explain body size misjudgments
Body checking task: fMRI task-based design<br>*Cross-sectional study*	Suda et al., 2013	AN = 20<br>HC = 15	DSM-IV	15.7 (1.0)<br>27.0 (7.5)	Neural correlates of body checking perception	AN reported higher anxiety compared to HC during the body checking task. The level of anxiety positively correlated with body shape concern scores. AN had less activation in the medial prefrontal cortex and right fusiform gyrus compared to HC in response to body checking and compared to neutral action images. Body shape concern scores correlated negatively with medial PFC activation in AN group.
Social and Physical Identity Appraisal Task: fMRI task- based design<br>*Cross-sectional study*	McAdams & Krawczyk, 2014	AN-WR = 18<br>HC = 18	DSM-IV	19.8 (1.6)<br>26.1 (6.8)	Investigate neural differences in social and physical identity	Strong impairments in PrC and vACC during the social identity evaluation in AN, with activation of other regions (MFG/dACC) to compensate self- perspective social judgments. No differences in the physical appraisal

					processing	conditions.
Social Identity Appraisal – V2 and Face Task: fMRI task-based design<br>*Cross-sectional study*	Xu et al., 2017	AN-WR = 24<br>HC = 18	DSM-IV	22.8 (2.7)<br>29.6 (8.1)	Examine neural correlates in processing self-stimuli in acute and weight-restored state	Differences in MPFC and seed salience network regions, as well as in social cognition network in AN-WR: social cognition and salience networks as biological traits in WR state.
Affective touch (skin stroking and skin indentation simulation tasks): fMRI task based design<br>*Cross-sectional study*	Davidovic et al., 2018	AN = 25 (4 AN-b/p + 21 AN-r)<br>HC = 25	DSM-IV	16.3 (1)<br>20.33 (2.2)	Cortical processing of affective touch and body image perception	No differences in primary tactile regions in AN. Less activity in the left caudate nucleus, bilateral lateral occipital cortex and abnormal functioning of dorsal striatum. Disturbed body image perception in AN.
Weight estimation task and body comparison task: fMRI task-based design<br>*Cross-sectional study*	Kodama et al., 2018	AN - WR = 12 (7 AN-r + 5 AN-b/p)<br>HC = 13	DSM-IV	20.7 (0.71)<br>33.2 (0.08)	Neural correlates of body comparison and weight estimation	In AN, significant greater activation in the pregenual anterior cingulate cortex while comparing their bodies with underweight female bodies. Smaller activation in the middle temporal gyrus corresponding to EBA when comparing their own bodies irrespective of weight. Inability of negative emotion regulation in response to body image, which persists after recovery.
Social tapped reward task (smiling faces and full human figured varied in attractiveness and weight): fMRI task-	Sweitzer et al., 2018	AN –WR = 20<br>HC = 24	DSM-5	22.48 (4.54)<br>22.50 (3.65)	Neurobiology of social reward evaluation in body image	The rewarding value of full body images decreases with a sustained disorder course (not starvation). Striatal differences related to social proficiency, which contribute to reduced reward activation. Social images with bodies

based design<br>*Cross-sectional study*					perception	are provocative of eating disorder symptoms: the drive for thinness inherent in the disorder interferes with the ability to experience the bodies of others as rewarding. Body images are experienced as less rewarding because of these maladaptive and distancing self-comparison processes that may become more entrenched the more stable the disorder course. Decreased medial prefrontal cortex activation in AN.
Video clips of own bodies and others: fMRI task based and r-fMRI designs<br>*Cross-sectional study*	Via et al., 2018	AN-r = 20<br>HC = 20	DSM-IV-TR	16.94 (1.26)<br>28.40 (9.30)	The role of the DMN in self and other body perception	Hyperactivation of the dorsal posterior cingulate cortex during own-body processing but a response failure to another's body processing at the precuneus and ventral PCC, in AN. Increased task-related connectivity was found between dPCC-dorsal anterior cingulate cortex and precuneus-mid-temporal cortex. AN patients showed decreased resting-state connectivity between the dPCC and the angular gyrus. The PCC and the precuneus are suggested as key components of a network supporting self-other-evaluative processes implicated in body distortion, while the existence of DMN alterations at rest might reflect a sustained, task-independent breakdown within this network in AN.
Perceptive and an affective body image task: fMRI task-based design<br>*Cross-sectional study*	Horndasch et al., 2020	AN adults = 19<br>HC adults = 17<br>AN	ICD-10	Adults<br>16.06 (2.04)<br>26.27	Neuronal affective and perceptive processing of body	Mainly differences of activation in insula and caudate between AN - HC. During a perceptive task, diminished activation of regions involved in perceptive and evaluative functions as well as emotional reasoning in AN.

		adolescents = 15<br>HC adolescents = 18		(6.71)<br>Adolescents<br>n.r.<br>15.72 (1.93)	image	During an affective task there was a tendency towards activation differences reflecting reduced ability of size estimation and impaired integration of visual and body perception with emotions.
Matching task using unaltered or spatial frequency filtered photos of others' bodies<br>*Cross-sectional study*	Moody et al., 2021	AN-WR = 20<br>BDD = 23<br>HC = 21	DSM-IV	20.4 (1.4)<br>23.3 (3.3)	Brain activation and connectivity patterns during body image	Hyperconnectivity in the dorsal visual network and hypoconnectivity in parietal networks compared with controls. Activity and/or connectivity were associated with symptom severity and appearance ratings of others' bodies.
Autobiographical memory task: fMRI task-based design<br>*Cross-sectional study*	Terhoeven et al., 2021	AN = 29 (21 AN-r + 8 AN-b/p)<br>HC = 30	DSM-5	15.1 (1.3)<br>23.2 (4.9)	Exploring neural correlates behind food/body related AM recall	Increase of AM recall for AN salient ED stimulus: reduced involvement of self-referential processing regions (emotional avoidance).
Morphing technique: EEG study (ERPs)<br>*Cross-sectional study*	Henn et al., 2022	AN = 22<br>HC = 22	DSM-IV	16.14 (2.63)<br>27.36 (12.26)	Psychological, cognitive, behavioral, and emotional key facets of body size estimation	A significantly earlier increase in SSVEPs (steady-state visual evoked potentials) emerged in AN compared to HC, with AN evaluating their bodies in the morphing process as big at a significantly thinner body size. AN showed faster reaction times in the categorical evaluation of body stimuli.
Quantitative dense-array EEG recording analysis method -	Susta et al., 2022	AN = 23<br>HC = 21	DSM-5	14(n.r.)<br>22(n.r.)[2]	Could qEEG predict treatment	Specific pattern of brain activation in response to body images in AN, that likely contributes to social-cognitive and

2 Median, not mean value.

Brain Activation Sequences (BAS): facial expressions and body images *Cross-sectional study*					response in AN?	behavioral impairments in anorexia. In addition, the substantial difference in the pattern of brain activation within the participants with AN and its association with treatment resistance deserves special attention because of its potential to develop a clinically useful prediction tool and identify potential targets for neuromodulator treatments and/or individualized psychotherapy.
Body image morphing technique: fMRI task based design *Cross-sectional study*	Karakuş et al., 2023	AN-r = 12 MDD = 9 HC = 10	DSM-5	17.4 (2.2) 14.8 (1.5)	Comparison between neural correlates of body image disturbance between AN and MDD	Increased activations in the parietal cortex, cingulate gyrus and parahippocampal cortex in response to underweight images in AN versus HC and MDD. No differences between groups regarding overweight images.
Body Size Estimation Task: EEG/MEG study *Cross-sectional study*	Rahder et al., 2023	AN-r = 36 HC = 42	DSM-5	15.60 (1.34) 15.33 (1.65)	Visual misperception hypothesis testing	Body image disturbances in adolescent restrictive-type AN patients depend on self-reference and do not represent a deficit of visual perception, but rather biased emotional attention.

Table 3. Classification of behavioral and neuroimaging studies on the multidimensional model of body image (1973 - 2023)

Label	Study	Component classification			
		Perceptive	Affective	Cognitive	Somat.
A	Slade & Russell, 1973	**	-	-	-
A	Garfinkel et al., 1978	**	*	-	-

A	Pierloot & Houben, 1978	**	-	-	-
A	Casper et al., 1979	**	-	-	-
A	Garfinkel et al., 1979	**	*	-	-
A	Strober et al., 1979	**	-	-	-
A	Meerman, 1983	**	-	-	-
A	Touyz et al., 1984	**	-	-	-
A	Fichter et al., 1986	**	-	-	-
A	Gardner & Moncrieff, 1988	**	-	-	-
A	Whitehouse et al., 1988	**	-	-	-
A	Penner et al., 1991	**	-	-	-
A	Probst et al., 1992	**	-	-	-
A	Molinari, 1995	**	-	-	*
A	Baluch et al., 1997	**	-	*	-
A	Lautenbacher et al., 1997	**	-	-	-
A	Gila et al., 1998	**	-	-	-
A	Hermans et al., 1998	-	-	**	-
A	Sackville et al., 1998	-	-	**	-
A	Hennighausen et al., 1999	**	-	*	-
A	Smeets et al., 1999	**	-	*	-
A	Smeets, 1999	**	-	-	-
A	Epstein et al., 2001	-	-	**	**
A	Smeets & Kosslyn, 2001	**	-	*	-
A	Fassino et al., 2002	-	-	**	-
B	Seeger et al., 2002	**	**	-	-

B	Wagner et al., 2003	**	-	*	-
A	Roy & Forest, 2007	**	-	*	-
A	Urdapilleta et al., 2007	**	*	*	-
B	Sachdev et al., 2008	**	-	*	-
A	Cserjési et al., 2010	**	**	*	-
A	Guardia et al., 2010	*	-	-	**
B	Fladung et al., 2010	**	-	*	-
B	Friederich et al., 2010	**	**	-	-
B	Mohr et al., 2010	**	**	-	-
B	Nico et al., 2010	**	-	-	-
C	George et al., 2011	**	-	**	-
A	Keizer et al., 2011	*	-	-	**
A	Guardia et al., 2012	*	-	-	**
B	Pruis et al., 2012	*	**	**	-
A	Urgesi et al., 2012	**	-	**	-
C	von Wietersheim et al., 2012	**	-	*	-
B	Castellini et al., 2013	**	**	*	-
A	Keizer et al., 2013	-	-	-	**
B	Suchan et al., 2013	**	-	-	-
B	Suda et al., 2013	**	**	*	-
A	Keizer et al., 2014	-	-	-	**
B	McAdams & Krawczyk, 2014	-	-	**	-
A	Øverås et al., 2014	**	-	**	-
C	Pinhas et al., 2014	**	-	**	-

C	Reichel et al., 2014	**	**	-	-
A	Smith et al., 2014	**	-	**	-
A	Spring & Bulik, 2014	**	**	-	-
A	Urgesi et al., 2014	**	-	**	-
A	Hagman et al., 2015	**	*	*	-
A	Horndasch et al., 2015	**	-	*	-
A	Spitoni et al., 2015	**	-	-	**
A	Cazzato et al., 2016	**	-	*	-
C	Cornelissen et al., 2016	**	-	-	-
A	Keizer et al., 2016	*	-	-	**
A	Mele et al., 2016	**	-	*	-
C	Phillipou et al., 2016	**	-	*	-
C	Svaldi et al., 2016	**	**	-	-
A	Zopf et al., 2016	-	-	-	**
A	Moody et al., 2017	**	-	*	-
A	Mölbert et al., 2017	**	*	*	-
B	Xu et al., 2017	*	-	**	-
A	Brockmeyer et al., 2018	-	-	**	-
B	Davidovic et al., 2018	-	**	-	**
B	Kodama et al., 2018	*	**	*	-
A	Mergen et al., 2018	*	-	-	**
A	Mölbert et al., 2018	**	-	**	-
B	Sweitzer et al., 2018	**	-	**	-
B	Via et al., 2018	**	*	*	-

A	Kazén et al., 2019	**	-	**	-
A	Provenzano et al., 2019	**	**	**	**
A	Brockmeyer et al., 2020	**	-	**	-
C	Hartmann et al., 2020	**	-	**	-
B	Horndasch et al., 2020	**	**	-	-
A	Porras et al., 2020	**	**	**	**
A	Beckmann et al., 2021	-	-	-	**
A	Behrens et al., 2021	*	*	**	-
A	Borgers et al., 2021	*	*	**	-
A	Brown et al., 2021	**	**	**	**
A	Demartini et al., 2021	**	*	*	*
A	Hasenack et al., 2021	**	**	-	**
C	Knejzlíková et al., 2021	**	**	-	-
B	Moody et al., 2021	**	-	-	-
A	Ralph-Nearman et al., 2021	**	*	-	-
B	Terhoeven et al., 2021	*	**	**	-
C	Couton et al., 2022	**	**	*	-
A	Di Lodovico et al., 2022	**	-	-	-
B	Henn et al., 2022	**	**	**	-
C	Mahr et al., 2022	*	**	*	-
B	Susta et al., 2022	**	**	-	-
A	Ambrosecchia et al., 2023	*	*	*	**
A	Fusco et al., 2023	**	-	**	-
A	Gadsby et al., 2023	**	-	-	-

C	Horndasch et al., 2023	**	**	-	-
A	Jenkinson et al., 2023	**	-	**	-
B	Karakuş et al., 2023	**	*	-	-
A	Lakritz et al., 2023	**	-	**	-
A	Meneguzzo et al., 2023	**	-	**	*
A	Meregalli et al., 2023	*	-	**	**
B	Rahder et al., 2023	**	**	-	-
A	Radix et al., 2023	*	**	**	-
A	Schloesser et al., 2023	**	-	-	-

Legend: Label A (behavioral study); label B (neuroimaging study). Degree of involvement: ** (primary involvement); * (secondary involvement); - (no involvement). Brief description of each component: perception = visual detection, estimation and identification of the body's properties (size, weight, shape); affective = emotional states, emotional regulation/appraisal strategies developed towards own body (satisfaction/dissatisfaction rates, fear, discomfort, obsession, anxiety, sensitivity, interoceptive awareness); cognitive = beliefs, cognitive schema/representations, attitudes used to evaluate and being in a relationship with own body (e.g., drive for thinness), cognitive processes (e.g., memory, decision making, attention, appraisal); somatosensory/sensorimotor = somesthetic, proprioception, somatic and bottom-up motor functions related to own body sensory perceptions and motor schemas interplay. Physiological label: C.
